# Ramp-to-threshold dynamics in a hindbrain population controls the timing of spontaneous saccades

**DOI:** 10.1038/s41467-021-24336-w

**Published:** 2021-07-06

**Authors:** Alexandro D. Ramirez, Emre R. F. Aksay

**Affiliations:** Institute for Computational Biomedicine and the Department of Physiology and Biophysics, Weill Cornell Medicine, New York, NY USA

**Keywords:** Neural circuits, Brainstem

## Abstract

Organisms have the capacity to make decisions based solely on internal drives. However, it is unclear how neural circuits form decisions in the absence of sensory stimuli. Here we provide a comprehensive map of the activity patterns underlying the generation of saccades made in the absence of visual stimuli. We perform calcium imaging in the larval zebrafish to discover a range of responses surrounding spontaneous saccades, from cells that display tonic discharge only during fixations to neurons whose activity rises in advance of saccades by multiple seconds. When we lesion cells in these populations we find that ablation of neurons with pre-saccadic rise delays saccade initiation. We analyze spontaneous saccade initiation using a ramp-to-threshold model and are able to predict the times of upcoming saccades using pre-saccadic activity. These findings suggest that ramping of neuronal activity to a bound is a critical component of self-initiated saccadic movements.

## Abstract (web summary)

The neural mechanisms by which the brain initiates self-generated actions are unclear. The authors show, using population imaging and single-cell ablation, that ramp-to-threshold firing dynamics over multiple seconds is critical for the initiation of self-generated eye movements.

## Introduction

While much is known about the manner in which motor systems execute behaviors, relatively little is understood about the neural systems that help us decide upon and plan for particular actions, especially those actions that are spontaneous and self-initiated. Classical work in humans identified the presence of premovement signals in EEG recordings that slowly built-up for over a second in advance of a self-initiated action^1^. Work in animal models suggests that this macroscopic activity is reflective of neuronal firing patterns where rates increase ahead of spontaneous movement in a ramp-like manner^2–4^. Furthermore, such studies have shown trial-to-trial variation in the timing of the movement is associated with variation in the ramp rate, with a slower rise coupled to longer rise time, suggesting that action is initiated when neural activity ramps to a cell-specific threshold^3,5^. More generally, such ramp-to-threshold dynamics have also been found ahead of goal-directed movements^6,7^ and in decision-making tasks^7–12^, where the animal is responding expressly to some externally provided stimulus, leading to the proposal that there may be common principles underlying the generation of ramp-to-threshold activity associated with motor planning for both spontaneous and externally driven actions^13,14^. However, there are many basic questions unanswered about this ramp-to-threshold dynamic, especially in the spontaneous setting, not only regarding how it is generated, but whether it is preparatory, possibly facilitatory (but not essential), or perhaps suppressive^15–18^.

The potential role of ramp-to-threshold dynamics in action initiation has been of particular interest in the visuo-motor system. In this setting, such dynamics have been most often investigated in the context of a goal-directed action, the performance of a saccade after a cue is provided to a visual target. Of note, neurons in the lateral intraparietal cortex, frontal eye fields, superior colliculus (or optic tectum), and the pons show, in addition to a burst of action potential at the time of goal-directed saccade, a buildup in firing characteristic of ramp-to-threshold dynamics^19,20^. Lesions in midbrain and forebrain regions lead to delays in goal-directed saccade initiation, consistent with a role for pre-saccadic activity in planning and preparation for saccades^21,22^. However, it is unclear what the role of these dynamics might be in the generation of spontaneous, self-initiated saccades. Although ramping activity is present on cortical, collicular, and tectal neurons ahead of spontaneous saccades, removal of these regions does not alter spontaneous saccade performance and apparently timing in the dark^23–25^. This suggests that structures in the hindbrain could be involved in the initiation of spontaneous saccades, an obvious choice being the pons, but potentially as well the cerebellum, where recently neurons associated with self-timing of saccades to visual targets have been found^26^.

To better understand the neural systems underlying the initiation of spontaneous saccades, we turned to a model vertebrate, the zebrafish. Even in the larval state, this animal makes robust spontaneous saccades in the dark without training or priming^27^. In the larval and juvenile stages, the entire brain of the intact animal is optically accessible, enabling tracking of neuronal activity at cellular resolution and enabling perturbative strategies, such as targeted ablations^28,29^ that examine causality. These advantages are particularly relevant given the commonalities in visuo-motor anatomy and physiology between zebrafish and other vertebrates^30–33^. Early calcium imaging studies in the larval zebrafish during saccadic behavior have reported single-cell activities ranging from burst-like to fixation-like in rhombomeres 7 and 8^32,33^. A more recent imaging study looking at regional activity showed that such signals extend into rhombomeres 1–6, and found preliminary evidence for ramp-like activity ahead of saccades in a dorsal region of rhombomere 7^34^. However, only one of these studies was focused on spontaneous saccades in the absence of visual inputs^32^, inputs that could be a source of confounding signals. Furthermore, none of these studies could unambiguously report on the activity of individual neurons since calcium sensors were distributed throughout the cell, potentially introducing neuropil contamination.

We combined two-photon calcium imaging and single-cell perturbations to identify a population of neurons controlling the timing of upcoming saccades. Our combined imaging and perturbation study provides three notable findings. First, we generated a comprehensive map of neuronal activity patterns underlying spontaneous saccades and subsequent fixations—these maps are generated while animals are in the dark, ensuring that the signals are internally generated, and with a nuclear-localized calcium sensor, thus eliminating neuropil contamination. Second, we found neurons in the hindbrain whose activity rises above baseline in a direction-selective manner multiple seconds before the decision to saccade. Furthermore, the time and rate of rise of these cells is consistent with a ramp-to-threshold model, which can be used to predict saccade timing. Third, we found, through targeted, single-cell ablations, evidence implicating these cells in controlling the proper patterning of spontaneous saccades. These findings not only provide insights into the mechanisms controlling the choice of when to shift gaze, but also establish a new model system for understanding the neuronal processes underlying spontaneous, self-initiated actions.

## Results

**Fig. 1:**
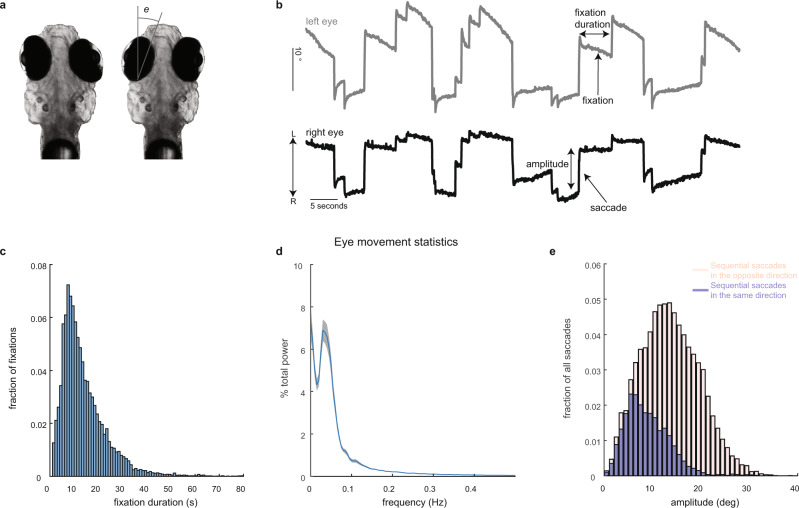
Spontaneous eye movements in larval zebrafish. **a** Changes in horizontal eye position, *e*, were recorded from agarose-restrained larval zebrafish while they made spontaneous eye movements in the dark. **b** Example changes in horizontal eye position with time for both eyes. Eye movements were quantified by examining saccade amplitude and fixation duration. The arrows labeled L/R indicate the direction of leftward/rightward movements. **c** Histogram of fixation durations measured across multiple animals (1 s bins; *n* = 16,033 fixations from 20 fish). **d** Power spectral density of eye position as a function of frequency. Data presented as mean (blue line) ± SEM (gray shading; *n* = 422 samples from 20 fish). **e** Saccade amplitude distribution measured in degrees (1° bins). We have separated saccades made in the same direction as the previous saccade (blue) from those made in the opposite direction (beige). Both histograms were normalized by the total number of saccades (*n* = 16,033 saccades from 20 fish). Source data are provided in a Source data file.

**Fig. 2:**
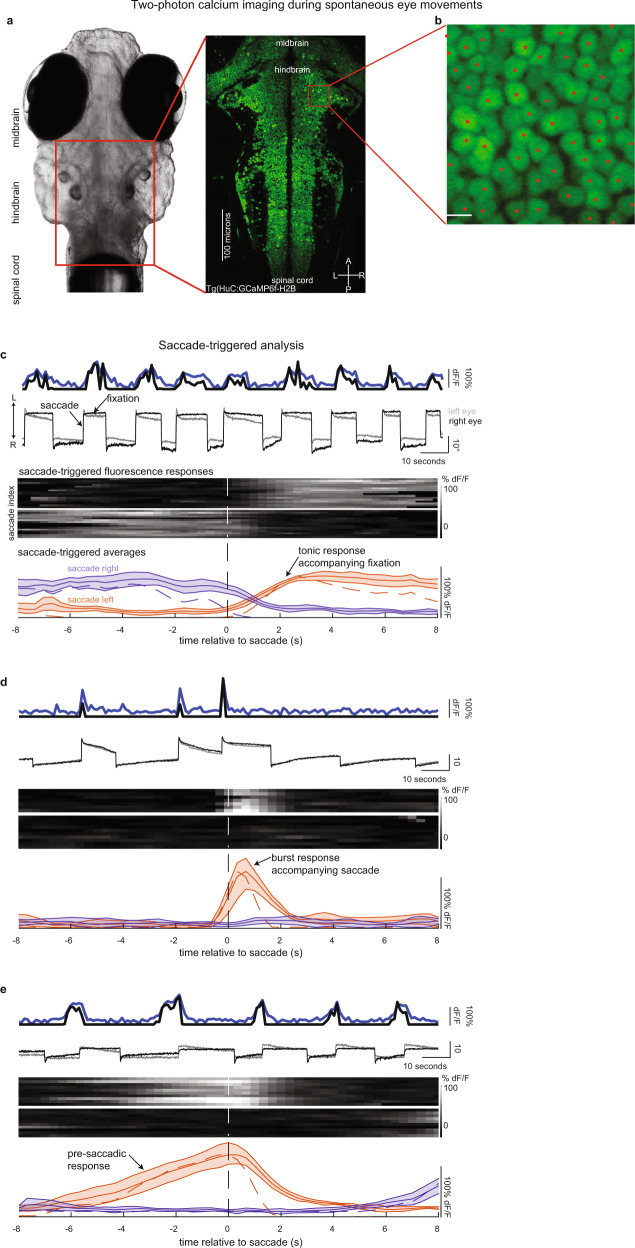
Two-photon calcium imaging during spontaneous eye movements. **a** Brightfield image of a 7-day-old larval zebrafish along with a time-averaged calcium image showing all neurons within a single plane of the hindbrain. A, P, L, and R denote anterior, posterior, left, and right respectively. **b** Zoomed-in image of time-averaged nuclear-localized GCaMP6f expression. Red dots show the center-of-mass for automatically detected nuclei from time-averaged fluorescence intensity images (see “Methods”). Similar results were seen across 422 planes collected from 20 fish. Scale bar length = 5 µm. **c**–**e** Examples of simultaneously recorded eye movements and calcium activity along with saccade-triggered responses and averages. (Top panel) simultaneously recorded left and right eye position (gray and black), single-cell fluorescence (blue), and deconvolved fluorescence (black) traces versus time. Deconvolved fluorescence is scaled to fluorescence traces and units are arbitrary. The arrows labeled L/R indicate the direction of leftward/rightward movements. (Middle panel) heatmaps of saccade-triggered responses around leftward (top map) and rightward (bottom map) directed saccades. Each row displays fluorescence versus time before and after each saccade. Dashed vertical line shows time of saccade. Source data are provided in a Source data file. (Bottom panel) saccade-triggered averages around leftward (red) and rightward (purple) directed saccades. Data are shown as average fluorescence (solid line) along with 95% confidence intervals (shaded region) and average deconvolved fluorescence (dashed line). **c** Example of a cell with tonic post-saccadic activity (*n* = 16 (15) samples for leftward (rightward) saccades). **d** Example of a cell that displays a burst of activity triggered during the saccade (*n* = 7 (9) samples for leftward (rightward) saccades). **e** Example of a cell that displays pre-saccadic activity (*n* = 9 (9) samples for leftward (rightward) saccades).

**Fig. 3:**
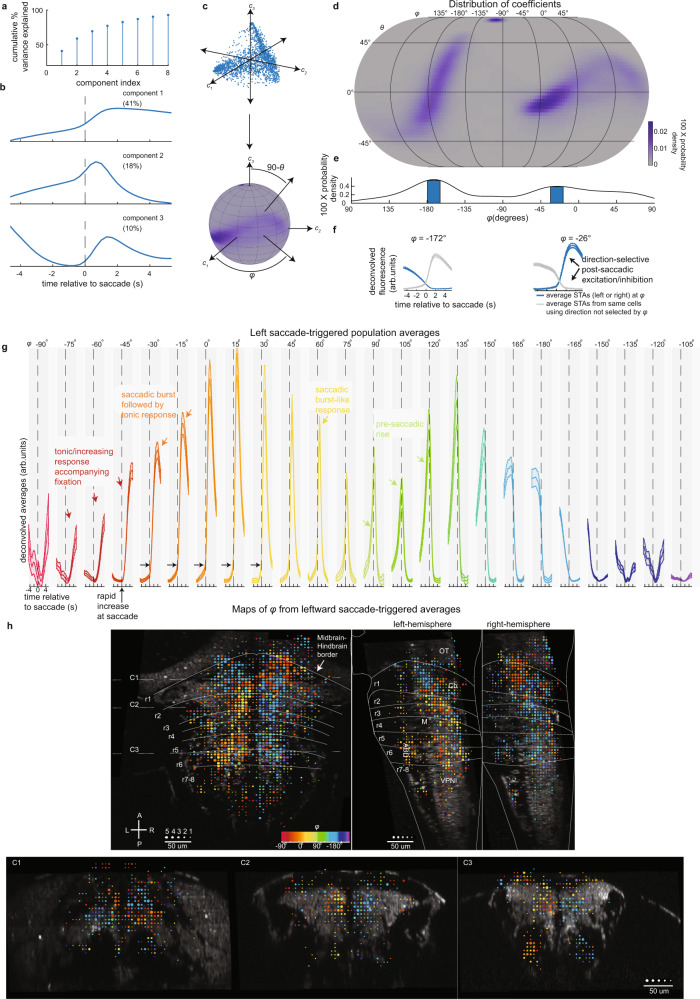
Functional and spatial distribution of neuronal responses. **a** Cumulative percent variance explained by each component found via principal component analysis on normalized saccade-triggered deconvolved fluorescence averages (STAs). **b** First three components sorted by percent variance explained (shown in the upper right). **c** Scatter plot of coefficient elements that scale components one (*c*_*1*_), two (*c*_*2*_), and three (*c*_*3*_). After unit normalization, we transform coefficients to spherical coordinates ($\varphi$ and $\theta$). **d** Eckert IV projection of coefficient probability density. **e** Probability density of $\varphi$ (shaded blue show peak locations). **f** Population average of STAs with $\varphi$ equal to peak locations in **e** ($\varphi =$ −26 ± 7.5 and −172 ± 7.5). Data shown as mean ± SEM (*n* = 1,553[932] cells examined over 16[16] fish at $\varphi =$ −26[−172]). **g** Series of population-averaged STAs triggered to saccades to the left with $\varphi$ equal to the value in the top row, (within 15°). Data shown as mean (solid line) ± SEM (shaded region). Varying number of cells: $\varphi =$ −90 (*n* = 41 cells, 14 fish), −75 (*n* = 51, 13), −60 (*n* = 171, 16), −45 (*n* = 426, 18), −30 (*n* = 488, 16), −15 (*n* = 407, 17), 0 (*n* = 291, 17), 15 (*n* = 297, 15), 30 (*n* = 278, 16), 45 (*n* = 269, 16), 60 (*n* = 246, 17), 75 (*n* = 266, 18), 90 (*n* = 134, 15), 105 (*n* = 91, 16), 120 (*n* = 122, 15), 135 (*n* = 135, 16), 150 (*n* = 212, 15), 165 (*n* = 402, 16), 180 (*n* = 292, 15), −165 (*n* = 864, 16), −150 (*n* = 373, 18), −135 (*n* = 53, 14), −120 (*n* = 49, 13), −105 (*n* = 351, 18). **h** Horizontal, sagittal, and caudal projections of a sample of cells used to construct (**g**) (*n* = 3,012 total cells examined over 18 fish). Color indicates most probable value of $\varphi$ for cells within 5 µm bins (using the same color scheme as **g**). Circle size indicates number of cells within bin (largest is $\ge$5 cells). Coronal projections are made within 30 µm of the dashed lines marked C1, C2, and C3 in the horizontal map. r rhombomere, OT optic tectum, Cb cerebellum, M Mauthner cell, VPNI velocity-to-position neural integrator, ABD abducens complex, L–R left–right, A–P anterior–posterior. Source data are provided in a Source data file.

**Fig. 4:**
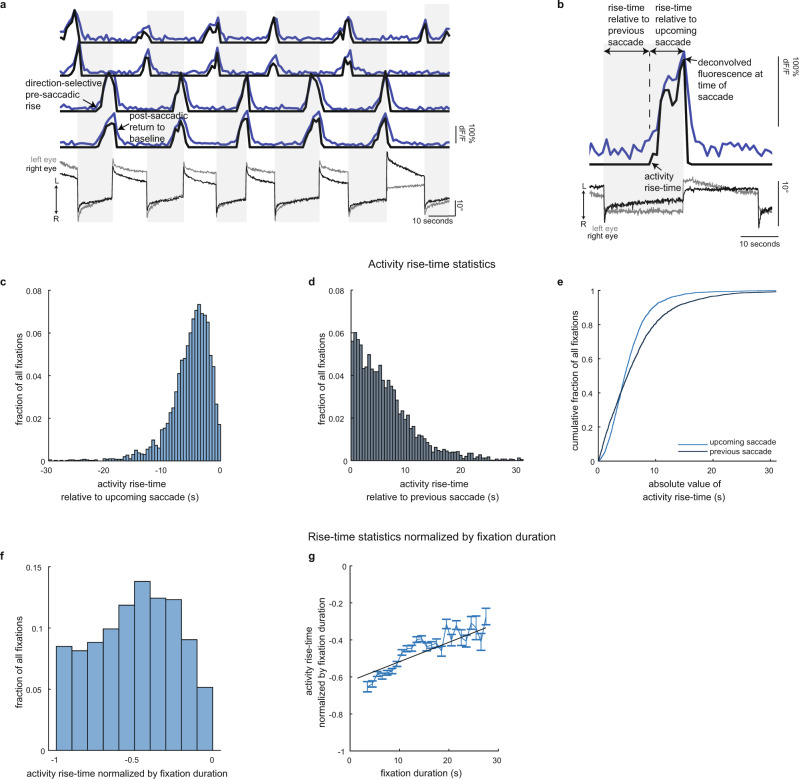
Neurons whose activity anticipates saccades in a direction-selective manner. **a** Simultaneously recorded eye position and single-cell fluorescence (blue) traces versus time from four cells whose activity rises before spontaneous saccades. Deconvolved fluorescence is overlaid in black and scaled to fluorescence (units are arbitrary). In **a** and **b**, the arrows labeled L/R indicate the direction of leftward/rightward movements. **b** Zoomed-in view of pre-saccadic fluorescence (blue) and deconvolved fluorescence activity (black) demonstrating the measurements used in subsequent analyses. **c** Histogram of pre-saccadic activity times of rise relative to the time of upcoming saccade. **d** Histogram of pre-saccadic activity times of rise relative to the time of the previous saccade. Bin size in **c** and **d** is 0.5 s. **e** Cumulative distribution functions of absolute value of pre-saccadic activity times of rise relative to upcoming or previous saccade. **f** Histogram of pre-saccadic activity times of rise, measured with respect to upcoming saccade, normalized by fixation duration (bin size is 0.1). **g** Pre-saccadic activity time of rise normalized by fixation duration as a function of fixation duration (blue line, 1 s bins). Data shown as mean ± SEM (*n* = 3,979 total fixations from 395 cells examined over 16 fish). Line of best-fit linear regression shown in black. Source data are provided in a Source data file.

**Fig. 5:**
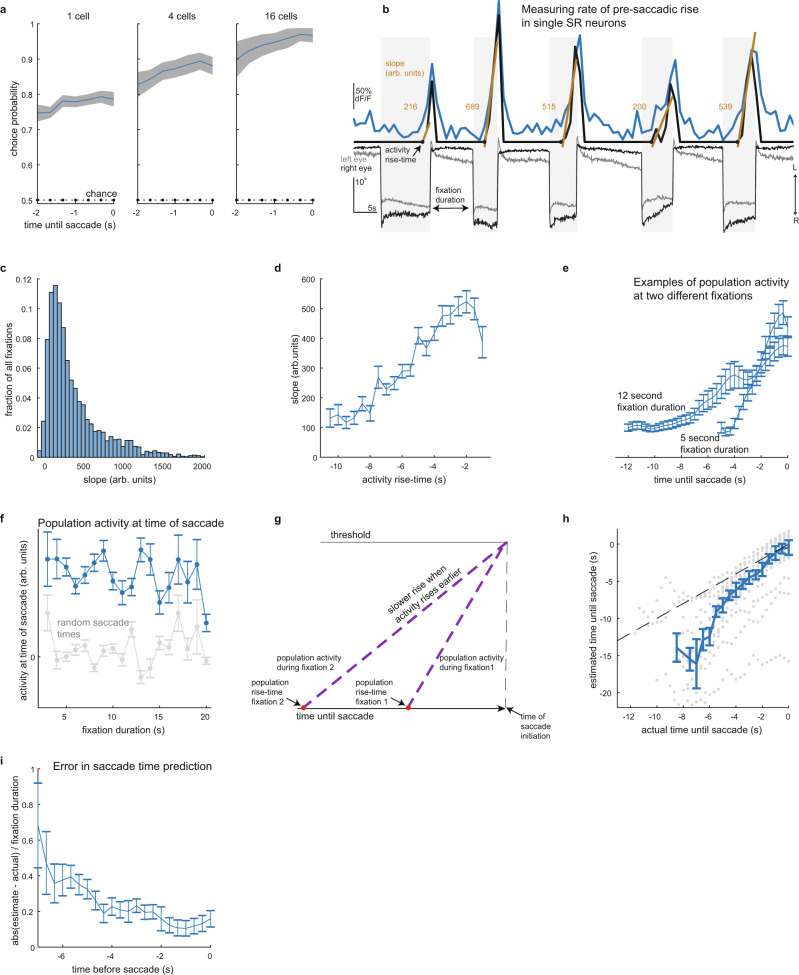
The rate and time of pre-saccadic rise vary in a manner consistent with ramp-to-threshold models. **a** Saccade direction choice probabilities. Data presented as mean (blue) ± SEM (gray) averaged over fixation duration (*n* = 19 samples examined over 16 fish). Each sample computed using 100 randomly selected fixations of constant duration. Dotted black line shows choice probability from random guessing. **b** Visualization of the slope statistic (gold, arbitrary units) used in **c** and **d** to quantify rate of pre-saccadic rise in deconvolved fluorescence before each saccade. Deconvolved fluorescence (black, scaled to fluorescence shown in blue) is plotted with simultaneously recorded eye movements. The arrows labeled L/R indicate the direction of leftward/rightward movements. **c** Histogram of slopes. Bin size = 50 (arbitrary units). **d** Slope versus time of pre-saccadic rise with respect to upcoming saccade (bin size = 500 ms; *n* = 2,715 fixations from 380 cells examined over 16 fish). **e** Example traces of population average, pre-saccadic deconvolved fluorescence as a function of time until upcoming saccade. Fixation duration was fixed (within 0.5 s) at either 5 s (*n* = 235 fixations from 107 cells examined over 10 fish) or 12 s (*n* = 168 fixations from 141 cells examined over 13 fish). **f** Deconvolved fluorescence (blue) at the time of saccade versus fixation duration (*n* = 2,375 fixations from 388 cells examined over 16 fish). Gray trace is from the same cells after selecting random saccade times. **g** Hypothetical mechanism for how a pre-saccadic signal could initiate saccades. **h** Predicted time until saccade versus actual time (gray dots). Blue line shows median ± SEM (500 ms bins; *n* = 405 samples from 388 cells examined over 16 fish). **i** Prediction error versus actual time until saccade. Data presented as median ± SEM (500 ms bins; *n* = 405 samples from 388 cells examined over 16 fish). Data in **d**–**f** are presented as mean ± SEM. Source data are provided in a Source data file.

**Fig. 6:**
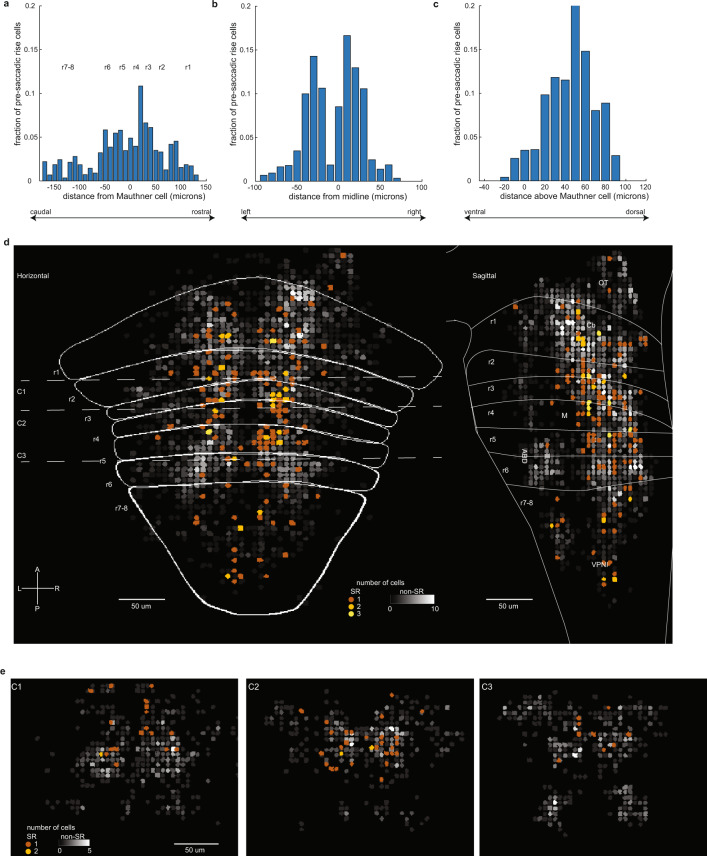
Spatial distribution of SR cells. Histograms showing fraction of SR neurons as a function of their registered location onto a common reference brain for the rostral–caudal (**a**), left–right (**b**), and dorsal–ventral (**c**) directions. Fractions are weighted to account for unequal numbers of fish used to sample different hindbrain regions. Bin size is 10 µm for **a**–**c**. Source data are provided in a Source data file. **d** Horizontal and sagittal projections showing the number of SR cells (*n* = 156 cells examined over 15 fish) and the number of eye-movement responsive cells that are not classified as SR (black-white color scheme, *n* = 2,945 cells examined over 18 fish). The number of cells displayed in each nonoverlapping, 5 µm bin was determined after subsampling to account for variations in the number of fish sampled per region (see “Methods”). r1–8 indicates approximate rhombomere location within the reference brain. OT optic tectum, Cb cerebellum, M Mauthner cell, VPNI velocity-to-position neural integrator, ABD abducens complex, L–R left–right, A–P anterior–posterior. **e** Coronal projections made within 30 µm of the dashed lines in **d** marked C1, C2, and C3.

**Fig. 7:**
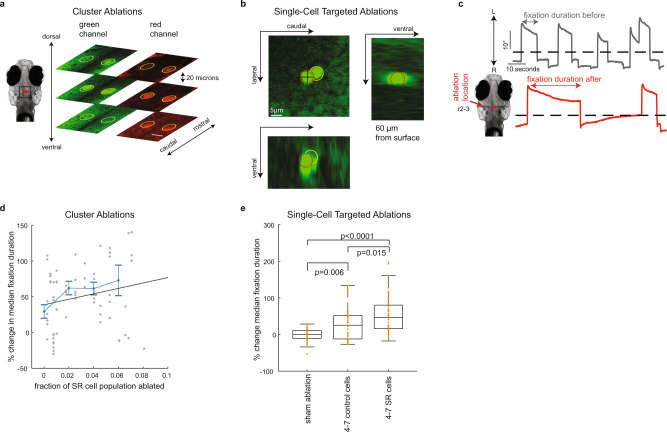
Focal laser ablations of SR neurons result in increased fixation durations. **a** Time-averaged images from an individual fish following bilateral laser ablations of clusters of cells. Ablated regions in each plane appear as bright, multi-spectrum fluorescent regions (outlined in yellow). Similar results were seen in 32 independent cluster ablation experiments. Scale bar is 30 µm. **b** Time-averaged images taken from an individual fish following a single-neuron ablation attempt (targeted cell outlined in red, off-target ablated cell outlined in yellow). Similar results were seen in 20 independent single-cell ablation experiments. **c** Example left eye position versus time recorded before (gray) and after (red) a bilateral cluster ablation was performed in the rostral hindbrain (r: rhombomere). The arrows labeled L/R indicate the direction of leftward/rightward movements. **d** Each gray point shows the percent change in median fixation duration following cluster ablation from a single animal (best-fit line shown in black). Blue line shows mean ± SEM of points (bin size = 2% fraction ablated; *n* = 82 points sampled from 29 fish; each point constructed with *n* = 57 samples, see “Methods”). Pearson correlation coefficient for the points shown here equals 0.19. **e** Percent change in median fixation duration following single-cell targeted ablations (individual samples shown in orange). For comparison, the boxplot labeled sham ablation shows percent changes in median fixation duration from non-ablated animals, using randomly selected fixations to form the sham before and after groups. Two-sample, two-sided, *t* test *p* values are displayed above boxplots. All *p* values displayed are significant at level 0.05 (using Bonferroni correction to control for family-wise error rate). Mean ± SEM percent change in median fixation duration equals 54 ± 8% (*n* = 44 from ten animals), 28 ± 7% (*n* = 40 from ten animals), −2 ± 3% (*n* = 40 samples from 40 animals) following SR, control, and sham ablations. Central line shows the median, box limits show the 25th and 75th percentiles, whiskers show 1.5 × interquartile range about upper and lower quartiles. Source data are provided in a Source data file.

In the first part of the results, we present an analysis of spontaneous eye movements in the dark in larval zebrafish (Fig. 1) and a map of the temporal dynamics of neurons that were active during this behavior (Figs. 2 and 3). We then analyze the temporal properties and spatial distribution of a special class of neurons found in this map whose activity suggests they play a role in eye movement initiation (Figs. 4–6). We end by systematically mapping how eye movement patterning is affected by ablations of these neurons (Fig. 7).

### Larval zebrafish generate spontaneous eye movements with a range of fixation durations

Larval zebrafish reliably generated spontaneous eye movements consisting largely of a back-and-forth alternation of rapid eye movements known as saccades followed by longer periods of relatively constant eye position known as fixations (Fig. 1a, b). Spontaneous eye movements in the horizontal plane were measured in agar-mounted larval zebrafish (*n* = 20 fish; 7–8 days post fertilization) after releasing the eyes from the agarose (Fig. 1a). Experiments were performed in the dark to ensure that movements were self-initiated and not occurring in response to a visual target. The time between saccades, known as the fixation duration (see Fig. 1b), was variable; fixations lasted between 2 and 50 s (1st and 99th percentiles) with a median duration of 11.4 s (*n* = 16,033 fixations; Fig. 1c). While there was a clear alternating cycle of leftward then rightward directed saccades (Fig. 1b), we found that it was not uncommon for eye movements to deviate from this simple pattern, as nearly a quarter of the time zebrafish made successive saccades in the same direction (22.9 ± 0.1%, average ± SEM across fish). To further examine the rhythmicity of these movements, we performed a Fourier analysis of the changes in eye position with time and found that power was distributed over a range of frequencies, with 95% of total power between 0 and 0.35 Hz, and peak power (excluding 0 Hz) at 0.03 Hz (Fig. 1d). The total range of eye position angles was 20 ± 1° (average ± SEM across fish of the 1st and 99th position percentiles averaged across both eyes). The size of saccades, known as the saccade amplitude (see Fig. 1b), varied between 2° and 28° (1st and 99th percentiles, median amplitude = 12°) and depended on the direction of the previous saccade (Fig. 1e; median amplitude of saccade in the same direction as previous saccade equaled 8°; median amplitude of saccade in the opposite direction equaled 13°). These results show that larval zebrafish can self-initiate a simple yet varying pattern of horizontal eye movements.

### Mapping activity using two-photon microscopy

To create a comprehensive map of the functional cell types involved in self-initiated eye movements, we imaged calcium dynamics throughout the larval zebrafish hindbrain (Fig. 2a) during spontaneously generated saccades and fixations in the dark. The seconds-long fixation durations between eye movements (Fig. 1b, c) facilitated the use of calcium imaging to analyze changes in single-cell activity during fixations. Activity from single neurons expressing nuclear-localized GCaMP6f under the control of the HuC pan-neuronal promoter was measured with raster-scanning two-photon microscopy (Fig. 2b). In each fish, we imaged a portion of the hindbrain using a stack of 5–68 horizontal planes. Stacks from individual fish were then registered to a reference brain (Supplementary Fig. 1a and see “Methods”). The planes were centered at different rostral–caudal locations across fish (imaging 20 fish in total) so that when combined via registration we effectively sampled responses from the entire hindbrain (Supplementary Fig. 1b), with each voxel sampled from at least 3 fish (Supplementary Fig. 1c)

We found that approximately a quarter of the neurons in the hindbrain showed some activity under these conditions. We used standard computer vision algorithms that detected cell locations based on local maxima in time-averaged fluorescence intensity images (see “Methods”) to count the total number of cells sampled (Fig. 2b and Supplementary Fig. 1d show cells detected via this method). We measured 238,191 hindbrain cells from all planes and fish in our dataset. This number is larger than the 120,000 neurons expected from sampling three complete hindbrains because some regions were sampled more than three times (Supplementary Fig. 1c). To infer how many of the total cells were active, we performed a separate analysis using the CaImAn algorithm which relies on nonnegative matrix factorization to identify the locations of responsive neurons^35^. We found that cells marked as background by the CaImAn algorithm generally had smaller peak fluorescence responses than non-background cells (Supplementary Fig. 1d, e). Considering these background neurons as inactive, we determined that approximately a quarter (62,896 hindbrain cells) of hindbrain neurons in our dataset were spontaneously active. In the rest of the manuscript, we ignore inactive cells and analyze cells identified by the CaImAn algorithm (see Supplementary Fig. 1f for a workflow of the analyses in the manuscript).

### Neuronal activity dynamics associated with spontaneous saccades and fixations

Approximately 19% of the spontaneously active hindbrain neurons had average activity related to eye movements. We aligned fluorescence and deconvolved fluorescence responses from active cells to the times of spontaneous saccades and determined which neurons had significant saccade-triggered average (STA) activity. We treated individual fixations surrounding a saccade as single trials (Fig. 2c–e and “Methods”) and performed a one-way ANOVA to compare the STA of fluorescence activity at different time bins to search for cells, with significant deviations from baseline. Significant STAs had clear changes in fluorescence (d*F*/*F*) and deconvolved fluorescence around or at the time of saccade (Fig. 2c–e). We will refer to cells with significant STAs as eye-movement responsive.

Eye-movement responsive cells had activity patterns that ranged from tonic activity during the post-saccadic epoch (Fig. 2c), to burst activity only at the time of saccade (Fig. 2d), to activity that gradually climbed during the pre-saccadic epoch (Fig. 2e). We used principal component analysis (PCA) to help represent how these activity patterns were distributed across the population. We found that 69% of the variance in normalized STAs of deconvolved fluorescence could be explained by three components (Fig. 3a, b). Therefore, we examined the distribution of coefficients along these components (Fig. 3c–e). The coefficients for the population were broadly, and mostly continuously, distributed (Fig. 3d, e).

A clustering analysis of the coefficients found that these data were best segregated into two clusters corresponding to left and right selectivity. The peaks in the density of coefficients (blue bars in Fig. 3e) corresponded to two distinctive STAs; one STA increased following saccades while the other STA decreased (Fig. 3f, blue traces). To measure how these patterns in STAs related to patterns in single-cell responses, we performed a *K*-means analysis on the combined coefficients for the two STAs, those around saccades to the left and right, that characterize single-cell responses. We found evidence that the coefficients for single cells were best segregated by two clusters (Supplementary Fig. 2a, b). The cluster means revealed that a typical cell has an STA that increases around saccades in one direction (left or right) and decreases around saccades in the other direction (Supplementary Fig. 2c). This direction-selective response captured the responses seen in most cells (see the cluster separation in Supplementary Fig. 2c and quantified in Supplementary Fig. 2b), indicating that the peaks in the density of coefficients (Fig. 3e) result from neurons that selectively respond to saccade direction.

We next generated population averages of STAs of deconvolved fluorescence grouped according to their coefficients along components 1 and 2 (see angle $\varphi$ in Fig. 3c and “Methods” section “Principal component analysis of saccade-triggered averages”) to ease visualization of both the dynamics across the population and the spatial distribution of the different activity profiles. STAs around saccades to the left were ordered by their value of $\varphi$, grouped into bins that are 15° in width, and then averaged together and plotted in Fig. 3g. The equivalent plot for saccades to the right is shown in Supplementary Fig. 3a. We registered the cell locations to a zebrafish brain atlas^36^ to map cell dynamics to neuronal locations (“Methods”).

We found stereotyped patterns of eye-movement-related activity at specific spatial locations. Many cells had pre-saccadic activity that remained constant at baseline levels, increased within a second of the saccade, and then displayed tonic firing after the saccade. Some of these neurons displayed primarily tonic firing after the saccade (e.g., $\varphi$ = −45 in Fig. 3g and Supplementary Fig. 3a); others had more burst-tonic characteristics (e.g., $\varphi$ = 0). Both cell types were broadly distributed in the hindbrain (Fig. 3h and Supplementary Fig. 3b). Prominent pockets of tonic and burst-tonic neurons with ipsilateral sensitivity were found in rhombomeres 7 and 8 in regions previously associated with the velocity-to-position neural integrator (VPNI in Fig. 3h), ventral portions of rhombomeres 5 and 6 associated with the abducens complex (ABD in Fig. 3h), dorsal regions of rhombomeres 5 and 6, and dorsal regions of rhombomeres 2 and 3; tonic and burst-tonic neurons with contraversive sensitivity were located in rhombomere 1 below the cerebellum. Other neurons had purely burst activity associated with ipsiversive saccades as expected for saccade generator cells ($\varphi$ = 60 in Fig. 3g and Supplementary Fig. 3a); these were most prominently clustered in ventral portions of rhombomeres 2 and 3. With increasing $\varphi$, we observed a pre-saccadic rise in activity that becomes longer in duration and more prominent relative to the burst event ($\varphi$ = 105 in Fig. 3g and Supplementary Fig. 3a). This pre-saccadic rise is suggestive of activity that is involved in the timing of upcoming saccades, a role which we will explore further in the remainder of this paper.

In summary, we found that ~5% of hindbrain neurons in larval zebrafish had responses associated with spontaneous saccades and fixations, these responses were direction-selective, and the response profile of this population was diverse. The diversity included cells with step-like profiles expected for ABD neurons and integrator neurons, and cells with burst-like responses expected for saccade generator neurons. The distribution also contained neurons whose activity is better described as anticipating upcoming movements, consistent with a role in saccade initiation, a role which we explore below.

### Single-trial analysis of cells with pre-saccadic rise activity

Within the continuum described above were cells whose population average activity steadily rose ahead of the upcoming saccade. Because this form of dynamics appears to anticipate a future movement, it is reasonable to speculate that neurons with such activity play an important role in saccadic preparation or timing. We now turn our attention to a closer examination of such neurons, with a focus on single-trial and single-cell level analyses that can capture variations missed with averaging.

We found that 6% (*n* = 401) of eye-movement responsive hindbrain neurons had fluorescent activity with clear ramp-like activity ahead of saccades (Fig. 4a). To systematically select these cells, we reanalyzed eye-movement responsive neurons (see workflow in Supplementary Fig. 1f) and determined which of these neurons had a Spearmen correlation coefficient between fluorescence activity and time to upcoming saccade significantly greater than zero (*p* < 0.01 using the Holm–Bonferroni method to correct for multiple comparisons). Here, we note some of the key qualitative features of these neurons before providing detailed quantification. These cells showed clear increases in activity before individual saccades either to the left (Fig. 4a, neurons in third and fourth row from top) or right (Fig. 4a, neurons in first and second row from top). For each cell, pre-saccadic rise events occurred ahead of saccades in one direction, although there were occasions where cell activity also rose before saccades in the opposite direction or failed to rise. d*F*/*F* for a small number of cells (*n* = 5) was significantly correlated with upcoming saccades in both directions. Given the small number of these cells we did not consider them in further analyses. Rise events generally lasted multiple seconds, and there was variability in the duration and rate of the rise both within and across cells. Further, for a given cell, activity tended to rise to consistent values at the time of saccade, even though duration of rise varied by 50% or more. We will refer to these cells as pre-saccadic rise (SR) neurons.

To quantitatively characterize the dynamics of SR cells, we assessed several features of their activity before and at the time of saccade that could elucidate their role in initiating the upcoming spontaneous saccade (Fig. 4b and “Methods”). To examine how the initiation of activity related to saccade occurrence, we measured the time when activity rose above baseline, and compared that to the time of upcoming and previous saccades. We also measured whether the time of activity rise scaled with fixation duration. To determine whether neuronal activity consistently rose to similar values, we measured activity at the time of saccade.

The initiation of the rise event was more tightly coupled to the timing of the upcoming saccade rather than the timing of the previous saccade. Across cells and fixations, activity rose over a range of times relative to the upcoming saccade (10th and 90th percentiles are 2 and 10 s respectively; median time = 5 s, *n* = 3,979 fixations across 16 fish; distribution shown in Fig. 4c). The timing of activity initiation when measured relative to the occurrence of the previous saccade was generally more variable, with a range of 1–13 s and with a variance (41 s^2^) that was nearly three times larger than the variance (14 s^2^) in timing when measured with respect to upcoming saccade (Fig. 4d). Furthermore, we rejected the null hypothesis that these two measurements in the time of rise come from the same distribution (Fig. 4e, *p* < 0.001; two-sample KS test). A similar trend was observed when data was analyzed on a per cell basis: 71% of cells had activity whose time of rise was more variable when measured relative to the previous saccade than when measured relative to upcoming saccade (ratios greater than one in Supplementary Fig. 4a). Therefore, the activity of SR neurons is more informative of upcoming saccades than previous saccades.

We found that cell activity did not rise at a fixed time relative to saccades. Given that there was notable variation in the fixation duration (Fig. 1c), we also tested for relationships between the time of rise and the duration of the fixation. To see if there was a constant scaling relationship between fixation duration and the time of SR activity rise, we examined whether the distribution of times of rise normalized by fixation duration was peaked (Fig. 4f). We found that, across cells and fixations, normalized rise times were not peaked at a single value, but rather distributed across the full range of possible values. The variability in normalized time of rise was related to fixation duration (Fig. 4g): there was a slight trend for activity to rise shortly after the previous saccade (normalized times near −1) during short fixation durations and for activity to rise later in the interval during longer durations (Fig. 4g best-fit line slope = 0.010 ± 0.001(1/s) and offset = −0.622 ± 0.007, estimate ± SEM). On a per cell basis, time of normalized rise and fixation duration appeared monotonically related for some cells but not others (see Supplementary Fig. 4b; 21% of cells had a Spearman correlation coefficient >0.5, see Supplementary Fig. 4c). While the distribution of correlation coefficients across cells was significantly different than shuffled controls (Supplementary Fig. 4c), within-cell variability made it difficult to discern significant linear trends (linear regression found 23% of cells had slopes with a *t* test *p* value <0.05, Supplementary Fig. 4d). In summary, we found that SR cell activity can rise within any fraction of the fixation time, with a propensity, at the population level, to begin rising later in the fixation for longer fixation durations.

### Pre-saccadic rise dynamics are predictive of upcoming saccades

If SR cell activity determines when a spontaneous saccade should occur, we should be able to predict whether a saccade is about to happen based on the output of SR populations. In this section, we show that SR pre-saccadic dynamics can be used to predict saccade direction and time. As part of this analysis, we also detail the relationships between the time at which an SR cell begins rising ahead of a saccade and the speed at which activity rises.

We first quantified how well SR cell activity can predict the direction of upcoming saccade. As an initial assessment of the information SR populations contain regarding upcoming saccade direction (left or right), we quantified population fluorescence using the choice probability (CP)^37,38^. The CP predicts saccade direction by comparing SR population average activity before saccades in the preferred direction with population activity before saccades in the non-preferred direction (see “Methods”). We found that CP, averaged across fixations, increased monotonically with time when aligned to saccade onset (Fig. 5a). Near the saccade, the CP was well above chance levels of 50% (Fig. 5a; mean CP = 77%, 87%, and 94% using single-cell activity or population averages consisting of 4 or 16 cells, respectively). For comparison, an ideal observer who has knowledge of the saccade transition probabilities would guess that an upcoming saccade is directed towards the opposite direction of the previous saccade and be correct 77% of the time (given that successive saccades occur in the same direction 23% of the time, see section Larval zebrafish generate spontaneous eye movements with a range of inter-saccade times*)*. Therefore, the CP performance is better than the performance of an ideal observer. These results indicate that spontaneous SR population activity contain information regarding the upcoming saccade direction.

We next examined the relationship between the time at which an SR cell began ramping and its rate of rise, finding that these dynamics generally fit under a ramp-to-threshold framework. For each cell and fixation, we measured the rate of rise of pre-saccadic activity (Fig. 5b and “Methods”). Across cells and fixations, the rate of rise of deconvolved fluorescence varied between −41 and 1,890 (arbitrary units, 1st and 99th percentiles, mean = 365, *n* = 2,715 events from 380 cells across 16 fish; distribution shown in Fig. 5c). This variability was positively correlated with the duration of ramping activity: slowly rising activity began to increase above baseline on average earlier than faster rising activity (Fig. 5d, e; at the individual cell level, this positive correlation was apparent for 24% of SR cells, Supplementary Figs. 4e and 5f). We also examined SR activity conditioned on whether two consecutive saccades occurred in the same or opposite direction (Supplementary Fig. 5a) and found similar trends in the distribution of the rate of rise (Supplementary Fig. 5b) and the correlation between rate of rise and duration of ramping activity (Supplementary Fig. 5c). Furthermore, the average activity reached similar levels at the time of saccade (Fig. 5e, f). These data suggest a picture where spontaneous saccades are triggered when the entire population of SR activity reaches a threshold, with rapid rates of rise associated with a shorter time to saccade initiation (Fig. 5g).

We next used the ramp-to-threshold framework to quantify how well SR neurons predict upcoming saccade times. We predicted saccade times by calculating when a ramp-to-threshold model, in which population activity growth is approximated as linearly increasing before saccade, passes a constant threshold level associated with saccade initiation (see “Methods”). We found good agreement between population activity and the ramp-to-threshold model when we trained the model using the entire period when SR activity increases (cc between model and data = 0.72, see Supplementary Fig. 6; 40% of cells were held-out for testing; 60% of cells were used to train the ramp rate). We next trained the model using a running estimate of the ramp rate, as one would do to predict saccade times in real time. We found that the predicted times to saccades and the actual times to saccade were well correlated (Fig. 5h, cc = 0.65 (*p* < 0.001)), even when we restricted our training window to the first 2 s after ramp initiation (cc = 0.53 (*p* < 0.001)). The timing error reached a peak performance near 20% and grew the farther back in time we attempted to predict saccades (Fig. 5i). By contrast, an ideal observer who has knowledge of the time of previous saccade and knowledge of the fixation duration probability distribution predicted saccade times with a timing error of 51%, and could not produce predictions that are correlated to the actual saccade times (cc = 0.00; see “Methods”). Thus, the pre-saccadic dynamics of SR population activity can be used to predict when saccades will occur.

### Spatial distribution of neurons with pre-saccadic rise dynamics

We next examined the spatial distribution of hindbrain cells with pre-saccadic rise dynamics. These neurons were broadly distributed across the rostral–caudal axis, with a small group in the cerebellum and rhombomere 1 (13%), the majority between rhombomeres 2 and 6 (77%), and a small group in rhombomeres 7–8 (10%; Fig. 6a). SR neurons were more likely to be near the midline than near the edge of the brain (Fig. 6b). Along the dorsal–ventral axis, SR cells largely resided in a region that is 10–70 µm dorsal of the Mauthner cell (Fig. 6c). None of the SR cells were found in the ABD complex (Fig. 6d), consistent with previous literature on ABD firing rate properties^39^. Directional preference of pre-saccadic neurons was largely (58%) contraversive (Supplementary Fig. 7), but we did observe clusters of ipsiversive preferring cells. The rostral–caudal distribution of SR neurons depended on the cell’s position along the dorsal–ventral axis; nearly all the pre-saccadic neurons located in the caudal portion of the hindbrain (rhombomeres 7–8) were contained near the Mauthner cell level or below (Fig. 6d).

### Focal laser ablations identify SR cells as indispensable for setting spontaneous fixation durations

It is unknown where the signals to initiate spontaneous saccade arise. The activity of the SR neurons we identified suggest that they initiate saccades when their population level activity reaches a threshold value. In the simplest instantiation of this model, activity is summed across cells and compared with a threshold value. If true, losses in the number of SR cells would lead to a longer time until saccades occur.

To test this possibility, we performed bilateral two-photon laser ablations at different locations in the hindbrain, where SR neurons are found and monitored changes in saccade and fixation metrics. We performed two sets of ablation experiments. In the first, we ablated cells that were clustered together in a volume that was approximately cylindrical in shape with a diameter of 30 µm along the rostral–caudal and medial–lateral axis and a side length of 60 µm along the dorsal–ventral axis (Fig. 7a). Within a single animal, two clusters (one on the left and one on the right hemisphere) were ablated at approximately the same rostro–caudal position (~1,200 cells, or 3% of the hindbrain, ablated per experiment). The rostro–caudal position of the cluster ablations was varied across animals. In the second set of experiments, we targeted single cells and removed 7–25 individual neurons based on their relationship to eye movements (Fig. 7b). In both sets of experiments, we measured changes in fixation duration following ablations (Fig. 7c).

We found that the fixation duration generally increased following cluster ablations in the hindbrain. Since it was not clear if hindbrain ablations would affect fixation duration, we first examined changes in fixation duration following ablations regardless of where in the hindbrain the ablation was performed. In a separate set of control animals, we performed similar size ablations in the spinal cord as a control for position-independent effects of ablation damage, such as heating or vascular injury. We rejected the null hypothesis that mean fixation durations were equal before or after ablations in the hindbrain or spinal cord (one-way ANOVA, *F* = 185.99, *p* < 0.01; distribution of durations shown in Supplementary Fig. 8a, mean fixation duration before and after ablations in the hindbrain equaled 11.9 and 17.9 s, respectively (*n* = 8,375 fixations before and 3,839 after from 26 fish); the mean fixation duration before and after ablations in the spinal cord equaled 13.2 and 12.9 s (*n* = 876 fixations before and 1,072 fixations after from 9 fish)), and we rejected the null hypothesis that the mean fixation duration before hindbrain ablations was equal to the mean after hindbrain ablations (difference in means and 95% confidence intervals equaled 6.0 [5.2, 6.8] s; *p* < 0.01, two-sample, two-sided *t* test using a Bonferroni correction for multiple comparisons). We also tested the effect of cluster ablations on saccade velocity and on the ability of animals to maintain fixation. We found a significant decrease in fixation stability following ablations in rhombomeres 7–8, consistent with previous literature^40^, but did not see a significant decrease in fixation stability after ablations in regions rostral of rhombomeres 7–8 (Supplementary Fig. 8b). We found no change in saccade velocity following hindbrain ablations (Supplementary Fig. 8c). These results provide evidence for a hindbrain role in spontaneous saccade initiation.

We found that the increase in fixation duration was correlated with the density of ablated SR neurons. We estimated the fraction of SR cells removed, for a given ablation, based on our map of their locations (Fig. 6) and plotted this fraction against change in fixation duration (Fig. 7d). The average Pearson correlation coefficient between the change in median fixation duration and fraction of SR cells equaled 0.26 (averaged across bootstrap samples from *n* = 10–29 fish, see “Methods”) and was significantly larger than randomly shuffled controls (*p* < 0.001, one-sided, two-sample KS test, *n* = 100 bootstrap samples using the Holm–Bonferroni method to correct for multiple comparisons). In a related analysis, we found that the fractional increase in fixation duration was largest for ablations in rhombomeres 3–4 (Supplementary Fig. 8d), where SR cells are prominently located (Fig. 6a, d). In conclusion, we found a weak, but significant correlation between increase in fixation duration and estimated fraction of ablated SR cells. To examine this issue more carefully, we measured the effect on fixation duration after ablating single SR neurons.

We performed single-cell targeted ablations and found a significant increase in fixation duration after ablating SR neurons. In each fish, we targeted 4–7 SR cells serially, resulting in the ablation of 7–25 cells in total since 1–4 cells nearby the targeted neuron were occasionally also ablated (see “Methods”). To examine nonspecific effects resulting from ablating a small number of cells, we targeted (in a separate control group of animals) four to seven neurons that were not eye-movement responsive, but were in the same region where SR cells were found. We then measured the percent increase in median fixation duration following ablation for each animal (Fig. 7e). We found SR-targeted ablations resulted in larger increases in fixation duration (45–66% [min–max], median = 56%, *n* = 100 bootstrap computations using repeated measurements from 10 fish, see “Methods”) than control-targeted ablations (22–39% [min–max], median = 29%) and found little evidence supporting the null hypothesis that the median effect size was equal between SR and control-targeted ablations (*p* values ranged from 0.0004 to 0.111, median = 0.007 from 100 one-sided Wilcoxon rank-sum tests with alternative hypothesis that the median effect size in SR ablations was larger than controls, see “Methods”). The relative increase in effect size in SR-ablated fish compared to control could not be explained by SR ablations affecting nearby (non-SR) eye-movement responsive cells because ablated SR cells were not closer than ablated control cells to (non-SR) eye-movement responsive cells (Supplementary Fig. 9). In summary, we observed an increase in fixation duration after ablating a small number of hindbrain neurons. The magnitude of the increase was largest when we specifically targeted SR cells. Both cluster and single-cell ablation experiments suggest that SR neurons play a significant role in the preparation for spontaneous saccades.

## Discussion

We combined focal laser ablations and calcium imaging to comprehensively map neuronal function and activity during a self-initiated behavior. We simultaneously measured eye movements and neuronal activity throughout the hindbrain of larval zebrafish, while they made spontaneous saccades in the dark. We discovered neurons in the hindbrain whose activity rises above baseline in a direction-selective manner multiple seconds before the occurrence of a saccade. We also implicated, through targeted ablations, a causal role for these cells in the decision to perform a spontaneous saccade. These data thus help elucidate the mechanism of a simple self-initiated behavior.

Our discovery of SR neurons in the hindbrain depended upon a comprehensive, single-cell resolution map of activity during spontaneous eye movements in the dark. Comprehensive spatial coverage was obtained by two-photon calcium imaging, which allowed us to image activity even at the deepest regions of the hindbrain where one-photon approaches suffer from poor resolution and signal-to-noise. Single-cell resolution of activity was ensured by coupling two-photon microscopy with nuclear-localization of the calcium sensor, allowing us to distinguish the activity profiles of even closely packed neurons. We maintained a focus on internally generated dynamics by monitoring activity while animals made spontaneous saccades in the absence of any visual cues. This effort allowed us to identify both a broad spatial distribution of the various signal types and strong regional characteristics, including a pronounced switch in the directional sensitivity of eye-position-related signals as one crosses from rhombomere 1 to 2, a clustering of burst neurons in the ventral portions of rhombomeres 2 and 3, and a high density of SR neurons in dorsal portions of rhombomeres 2 and 3.

These mapping results complement previous work in several ways. First, in relation to a mapping effort of spontaneous activity in the dark restricted to the caudal hindbrain^40^, the current map greatly extends spatial coverage but identified a somewhat sparser distributions of neurons with eye position signals in the caudal hindbrain. This greater sparsity could be related to differences in cooperativity of the calcium sensor employed. Second, in comparison to mapping work involving optokinetic behavior^41,42^, the results in this paper provide information on activity associated with the preparation for and initiation of spontaneous saccadic movements. Third, in relation to a mapping effort involving phototactic behavior and light-sheet microscopy^34^, we observed some similarities in the distribution of position signals (e.g., the pronounced reversal in sensitivity from rhombomere 1 to rhombomere 2), but several differences like a significant clustering of neurons with pre-saccadic ramp signals in dorsal portions of rhombomeres 2 and 3 (Wolf et al. presented preliminary findings of a region in dorsal rhombomere 7 where spatially averaged activity rises a few seconds before exhibiting burst). These differences might have arisen because of how the calcium sensor was distributed (nuclear versus cytoplasmic), strategies for signal detection (ANOVA STA-based versus position/velocity regression-based), and the challenging conditions for light-sheet microscopy in the hindbrain. Fourth, in comparison to work using locomotor behaviors to map hindbrain activity^43,44^, the current work suggests that regions in dorsal rhombomeres 2 and 3 that have been associated with the choice of which direction the body will turn are also associated with the choice of which direction the eye will turn. Given the tendency for eye movements to precede head movements in gaze control^45^, it will be of interest in the future to determine what role SR neurons play in orientation behaviors more broadly. Thus, our work, together with prior mapping studies, build a solid foundation for exploring and understanding how visual and volitional signals are combined and transformed into motor commands for oculomotor behavior and potentially other orientation tasks.

Are there common underlying causes and dynamics that trigger self-initiated actions? A great number of animal movements are initiated without external cues but their causes are unknown. There is tantalizing evidence, from crayfish to humans, that a buildup in neural activity is related to self-initiated movement^3,5,46–48^, but there has been no clear evidence that such neural activity is required for this type of behavior. Indeed, some experiments have suggested that buildup activity is only facilitatory, and other experiments have provided evidence that buildup activity is actually suppressive^16–18^. Our comprehensive mapping of this buildup activity enabled us to show a titration between the degree of disruption to the readiness signal and delays in the initiation of saccades. Moreover, single-cell ablations of SR neurons led to significant delay in saccade initiation that was two-fold greater than any delays following ablation of nearby non-SR cells. These results suggest that the spontaneous decision to move the eyes requires the buildup of activity in SR cells. Our work thus builds upon prior studies of signaling during voluntary movements by drawing a direct link between readiness signals and self-initiated movement.

The activity of SR neurons is consistent with that expected by ramp-to-threshold models for triggering behavior. In these models, a signal external to the ramp network is accumulated in value until a threshold is reached, at which point a command is given to trigger movement. While typically applied to psychophysics experiments, where the signal being accumulated represents evidence for a task-relevant decision^49–51^, the general features of such models, namely ramp rates that vary inversely with ramp times and consistent thresholds at which movement is initiated, are also applicable here. At the population level, we found that the rate of rise in SR neuron activity varied in a characteristic fashion (Fig. 5d), with longer ramp times closely tied to slower ramp rates so that, regardless of the fixation duration, population-wide pre-saccadic activity rose to a fairly constant value at the time of saccade (Fig. 5f). Furthermore, we were able to obtain reasonable predictions as to when an upcoming saccade would occur simply by extrapolation of the ramp trend established a few seconds after SR population activity was initiated (Fig. 5h). Our finding that ramp-to-threshold dynamics play a key role in the preparation for spontaneous saccades is consistent with the idea that, similar to decision-making tasks driven by accumulation of a signal representing external evidence^52,53^, spontaneous decisions like the one studied here involve a process where some internally generated signal that is external to the ramp network is accumulated^13,14^.

We assumed a linear transformation between calcium dynamics and firing rate. This assumption, over a limited range, is supported by previous work on the neural integrator^40^ and by the fact that a very strong nonlinearity would cause fluorescence signals to transition from off to on states, which would preclude us from seeing certain dynamical properties, such as approximate linear ramping activity of SR cells. However, weaker nonlinearities not accounted for in our analysis can cause discrepancies in our estimates of firing rate, particularly at low values and high values following saccades in burst and burst-tonic neurons. These discrepancies can also cause the slope and time of rise of SR cells to be artificially shortened from the actual times. However, the systematic nature of such nonlinearities would still allow us to observe the coarse dynamical profiles of cell responses and properties, such as the dependence of slope on time of rise.

The above arguments lead to three interesting questions. First, what might the internally generated signal(s) driving the ramp network be? Second, what is the mechanism that allows SR neurons to accumulate the signals they receive over time? Third, what might be the role of SR neurons in other saccadic behaviors, such as targeted saccades and fast phase eye movements? Although these questions will require much future work to answer, several hypotheses may be attractive to investigate. Regarding the first question, one interesting possibility arises from the observation that even though neurons with fixation-related activity were widely distributed, only ablations in the caudal hindbrain led to a leaky fixation deficit characterizing loss of functionality in the VPNI. This raises the possibility that the ongoing fixation-related activity in the rostral hindbrain could provide a kind of evidence signal that SR neurons accumulate. Regarding the second question, since the accumulation process is mathematically equivalent to temporal integration of a constant input signal, it is interesting to consider the possibility that SR neurons use a similar mechanism of recurrent excitation to promote integration as has been evidenced in the nearby VPNI for integrating saccadic inputs^54–57^. Regarding the third question, given that SR neurons are active much earlier than midbrain and cortical cells with pre-saccadic activity^11,51,58^ and given the widespread projections from the reticular formation to mid and forebrain areas^59–61^, it is possible that the SR signal in the hindbrain provides the initial kernel of activity needed to prepare movement during cued behaviors.

Answering these questions and understanding the circuit and cellular mechanisms underlying the seemingly universal ramping signal will require a combination of anatomy, perturbation experiments, electrophysiology, and mathematical modeling. One key advantage of discovering the SR signal in larval zebrafish is that in this animal one has the ability to image and manipulate single cells across the entire brain^62,63^, and perform whole-circuit connectomics analysis in functionally specified populations^54^. The mechanistic insights that one can obtain on readiness in larval zebrafish will hopefully allow us to understand a broad set of decision-making processes.

## Methods

### Calcium imaging and eye tracking

All experimental procedures were approved by Weill Cornell Medicine’s Institutional Animal Care and Use Committee. Transgenic larvae (7–8 days post fertilization) expressing nuclear-localized GCaMP6f, Tg(HuC:GCaMP6f-H2B; strain cy73–431), were kindly provided by Misha Ahrens’ lab. Fish were embedded in 1.5% low-temperature agarose and subsequently imaged using a custom-built two-photon laser scanning microscope (Daie, Goldman, and Aksay, 2015). We performed two-photon imaging using excitation light (930 nm) from a tunable laser (Spectra-Physics Mai Tai) sent through a 40× (0.8 NA) water-immersion objective lens (Olympus LUMPLFL40XW/IR2) to the hindbrain. The laser power was controlled using an electro-optical modulator (Conoptics 350-50UV) and amplifier (Conoptics 302RM). Laser power used for imaging ranged from 15–25 mW at the sample. Neurons within square horizontal planes (185 µm in length; 5–68 planes per fish spaced apart by 5 µm) were imaged simultaneously at 0.98 Hz (512 lines at 2 ms per line) by raster scanning implemented using ScanImage v3.5. Recordings lasted 4–5 min per plane. Images were saved as uncompressed grayscale TIFF stacks. Horizontal eye position (*e* in Fig. 1a) was extracted from camera images in real time at a variable sampling rate of ~13 Hz (refs. ^40,64^), using a substage CMOS camera (Allied Vision Technologies, Guppy FireWire camera) illuminated with infrared light (850 nm, Thorlabs 851 L). The strong contrast in intensity between the zebrafish eyes and the surrounding, transparent, area on the head (Fig. 2a) was used to determine pixels covering the eyes. At the beginning of each experiment, the experimenter manually selected (using *roipoly* in MATLAB) a region-of-interest about which the eyes were free to move. Pixels within the region-of-interest whose intensity values were below a manually selected threshold were classified as belonging to the eyes. The threshold was chosen during the experiment by manually trying various values and selecting the one that achieved the best segmentation quality. After threshold, two ellipses were fit to the resulting binary image using the MATLAB *regionprops* function. Eye position equaled the orientation of the fitted ellipse about its time-averaged value.

### Registration of individual planes to the Z-Brain atlas

Each imaging plane was first registered to a corresponding plane in a reference bridge brain constructed from a single Tg(HuC:H2B-GCaMP6f) fish (8 d.p.f.) using two-photon microscopy. Single planes in the bridge brain was constructed by stitching together six overlapping 512 × 512 (185 × 185 µm^2^ area) images through an optimized translation procedure using the FIJI Grid/Collection Stitching Plugin^65,66^. Each image used in the stitching procedure was formed by averaging calcium activity over a 20 s interval. The six images used were sufficient to completely tile any horizontal plane in the hindbrain. Bridge brain images were taken from across the entire extent of the hindbrain at a dorsal–ventral spacing of 3 µm. For each animal, we computed an affine transformation to linearly transform three-dimensional (3D) points from that animal to 3D points in the bridge brain. We used the *BigWarp* tool^67^ in FIJI to select *k* corresponding locations between the brain being registered and the bridge brain. Correspondence was determined by visual inspection based on features, such as fiber bundles, the midline, and ventricles (Supplementary Fig. 1a). We used the MATLAB “\” operator to solve the affine transformation ${\bf{y}}={\bf{Tx}}\,{\boldsymbol{+}}\,{{\bf{T}}}_{{\bf{0}}}{\boldsymbol{\otimes }}{\bf{1}}$ for the 3 × 3 matrix, ${\bf{T}}$, and the 3 × 1 translation vector ${{\bf{T}}}_{{\bf{0}}}$, where ${\bf{y}}$ and ${\bf{x}}$ are the 3 × *k* matrices of points chosen from the bridge brain and brain being registered respectively, ${\bf{1}}$ is a *k* × 1 vector of all ones and ${\boldsymbol{\otimes }}$ denotes the outer product. We repeated this procedure to find corresponding points and an associated transformation matrix between the bridge brain and the Elavl3-H2B brain (Elavl3 is another name for the HuC gene) available on the Z-Brain website.

### Image pre-processing to correct for movement artifacts

We were able to correct for small drifts that occurred during imaging using a motion-correction procedure based on cross-correlation. We first calculated the median fluorescence intensity across time for each pixel in the movie and used the resulting image as a reference. Any frame in the movie that deviated from the reference image was considered to have a movement artifact which required correction. To register each movie frame to the reference, we used the MATLAB function *dftregistration.m*^68^, which implements image registration via cross-correlation. The *dftregistration* algorithm estimates the peak in the two-dimensional cross-correlation between the reference image and movie frame being registered. Each movie frame is then translated by an amount determined from the peak location. For computational efficiency, the *dftregistration* algorithm works in Fourier space to calculate cross-correlations. We used MATLAB’s built-in fast Fourier transform software (*fft2*) to compute each frame’s two-dimensional discrete Fourier transform (DFT) and to compute the two-dimensional DFT of the reference frame.

### Detecting samples corrupted by animal movement

The motion-correction algorithm described above returned a scalar metric for each movie frame that indicated how well the frame matched the reference after correction. If this value was too low, the frame was considered too aberrant to be useful and the fluorescence of all pixels in this frame were replaced by NaNs. Specifically, for each frame, the *dftregistration* algorithm returned an error value related to the square root of one minus peak, normalized cross-correlation between a given frame and the reference. For each imaging plane, we computed the median error across all frames and the median absolute deviation (MAD) of the error across all frames. If a given frame’s error value was greater than five times the MAD plus the median that frame’s pixels were replaced by NaNs.

### Automated identification of active cells

We determined the locations of active cells by running the freely available CalmAn-MATLAB toolbox provided by the Flatiron Institute (https://github.com/flatironinstitute/CaImAn-MATLAB)^35^ on motion-corrected fluorescence movies. The algorithm models a calcium fluorescence movie as the product of two nonnegative matrices, one containing spatial locations and the other containing calcium time series for each active cell, plus a background and noise component. To determine the nonnegative matrices that best-fit the data, we implemented a procedure based on the *demo_script.m* file provided with the code. After initializing the spatial and temporal components, we ran one iteration of spatial and temporal updates, followed by a post-processing step, where components correlated with each other were merged and components that were poorly correlated with the data were removed, followed by a final spatial and temporal update.

We first found initial estimates for the spatial, temporal, and background components, using the *initialize_components* function. This function ran several steps: (1) it spatially filtered the fluorescence movies (Gaussian kernel with standard deviation set to 5, which corresponds to 1.8 µm). (2) It greedily selected locations where the spatial estimates explained the largest amount of spatio-temporal variance. (3) It used rank 1, nonnegative matrix factorization to produce spatial, temporal, and background estimates. (4) It refined these estimates using a hierarchical, alternating, nonnegative matrix factorization method. (5) It ran a rank 1 nonnegative matrix factorization on the spatio-temporal residual to initialize the background spatial and temporal components.

We updated the initial estimates of the spatial footprints and the background component, using the constrained nonnegative Lasso algorithm implemented in the *update_spatial_components* function. We used the *dilate* option which restricted the search of possible nonzero component values to a dilated version of that component’s nonzero values found in the previous iteration (dilation was performed using a 4-pixel radius (1.4 µm) disk-shaped structuring element). The new components are then post-processed by the following operations: (i) two-dimensional median filtering with a default size of 3 × 3 pixels, (ii) morphological closing with a square-shaped structuring element (3 pixels long), and (iii) “energy” thresholding with threshold set to 0.99. We then updated the estimates of the temporal components using the *update_temporal_components* function with an auto-regressive parameter, *p*, equal to zero. This function updated components using a block-coordinate descent algorithm (we used two iterations) which, with *p* equal to zero, ran a thresholding operation (at a threshold of 0) on the activity of each component after removing the effect of all the other components. After one spatial and temporal update, we removed spatial or temporal components that were poorly correlated with the raw data (space and time *r* values returned by *classify_comp_corr* function were <0.05) or whose spatial footprint areas were smaller than a value of 16 pixels squared which equaled 2.1 µm^2^. We then merged spatially overlapping components with highly correlated temporal activity (cc > 0.95), using the *merge_components* function.

Using the new component estimates, we then ran one more iteration of the spatial component update followed by one more iteration of *update_temporal_components*, however *p* was now set to one instead of zero. When *p* > 0, the temporal update deconvolves the activity of each component after removing the effect of all the other components. We used the default *constrained_foopsi* method along with the CVX toolbox for deconvolution, which solved a noise-constrained optimization problem to produce estimates of denoised fluorescence activity and nonnegative spike estimates. We estimated the noise level for each neuron by averaging the power spectral density of component fluorescence activity over high frequencies (one half the Nyquist frequency to the Nyquist frequency which equaled 0.24–0.49 Hz for our data). We set the auto-regressive parameter to a fixed value, equal to 1.3 s in the equivalent continuous time model, which we chose by measuring the rate of decay of putative VPNI cells when they transition from spiking to quiescence. Simultaneous electrophysiological and calcium recordings of VPNI cells from previously performed experiments have shown that such a procedure can be used to accurately estimate the auto-regressive parameter^40^. VPNI cells were selected as cells in rhombomeres 5–8 whose Pearson correlation coefficient between fluorescence and eye position was reasonably high (>0.5). As an initial approximation of the effects of calcium buffering on the relationship between firing rate and eye position, we convolved the eye position using an exponential decay kernel with a 1-s decay time before measuring the correlation. We then fit a decaying exponential function ($A{e}^{-t/\tau }+b$) to the average fluorescence triggered around nasally directed saccades made by the eye ipsilateral to the cell. $b$ was chosen as the average value of the ipsiversive STA 1–2 s prior to saccade. $A$ and $\tau$ were found by minimizing the squared error between the model and data using an interior-point algorithm (MATLAB *fmincon)* with $\tau$ constrained to be positive and $A$ constrained to be larger than $b$. We used the median value of $\tau$ from cells that were well fit by the exponential decay model (*r*^2^ > 0.8), as the parameter for the auto-regressive model (we converted the value from seconds to the equivalent discrete-time model). We used the nonnegative, sparse deconvolved output from this temporal update as our estimate of deconvolved neural activity. We estimated each component’s noisy fluorescence activity as the trace that resulted after spatially averaging the fluorescence video using that component’s spatial values as weights.

We investigated different initial values of the number of neurons parameter, *K*, in function *initialize_components*, and chose a value (250) that produced reasonable looking spatial footprint estimates based on visual inspection of a handful of sample fluorescence movies. Footprints in our final dataset had a size of 14.9 ± 0.1 µm^2^ (mean ± SEM; *n* = 62,896 cells examined over 20 fish), which aligned closely with the typical area of the nucleus visualized by H2B-GCaMP (Fig. 2a). We registered each cell to a reference brain (see “Methods” section “Registration of individual planes to the Z-Brain atlas”) and excluded cells that were registered to the midbrain.

### Cell detection based on time-averaged intensity images

In the “Results” section (“Mapping activity using two-photon microscopy”), we give estimates of the total number of cells in our dataset, and in Supplementary Fig. 1d, e we refer to non-active cells that were not selected by the CalmAn algorithm. For these sections only, we analyzed time-averaged images to infer cell locations since the CaImAn algorithm cannot find non-active cells (non-active cells are included as part of a single background term).

For each motion-corrected fluorescence movie, we calculated the median intensity across time for each pixel and analyzed the resulting time-averaged image to find individual cell nuclei locations. We performed a morphological opening on the time-averaged image (MATLAB function *imopen*) with a disk-shaped structuring element that had a radius equal to 4 pixels (1.4 µm). The opening operation with this structuring element tended to make it easier to segregate the disk-shaped nuclei in the image. We then found local intensity maxima of the opened image by looking for connected pixels with equal intensity that were greater than the intensity of external boundary pixels (MATLAB function *imregionalmax*). We measured the locations of individual cell nuclei measuring the regions of connected pixels that corresponded to local intensity maxima. To control for false positives, we excluded any regions-of-interest that had an area greater than most cell nuclei areas, which we determined by manual measurements (18.7 µm^2^ which translated to 144 pixels squared).

### Automated determination of saccade times

We determined the times of saccade occurrence by calculating the crossing times of eye velocity past a threshold. To calculate eye velocity, we first filtered out fluctuations in eye position using a median filter (*medfilt1* in MATLAB). The exact value of the filter order depended on the eye position sampling rate, but was chosen to correspond to 500 milliseconds. We then approximated eye velocity as the difference in filtered eye position at consecutive time points divided by the time difference between these points. The threshold was set to three standard deviations above the mean-absolute velocity or 10° s^−1^, whichever was larger. A single saccadic event typically consisted of several consecutive points whose velocity was above the threshold. We took the initial point as the time when the saccade occurs. During head/body movements the eye position traces become corrupted. One signature we used to determine when head/body movements occur is the time between threshold-crossing events (this signature was used in combination with the criteria listed in “Methods” section “Detecting samples corrupted by animal movement”). Separate experiments with video recordings of the entire body suggested that unusually short intervals between events typically indicate that the events occur during sudden head/body movements. For this reason, we did not consider threshold-crossing events that were spaced apart in time 1.4 s or less to be saccades.

### Saccade-triggered average calculation

We averaged saccade-triggered signals across fixations after linearly interpolating them to a grid of time points equally spaced apart by 1/3 s. Activity before and after each saccade was extracted, interpolated (using *interp1* in MATLAB with the *linear* method), grouped across saccades and then averaged according to the direction of saccade (left or right; Fig. 2c–e). We performed this procedure using deconvolved fluorescence and d*F*/*F*, where d*F*/*F* was computed as raw fluorescence, $F$, subtracted and divided by its mean across the entire recording period, ${F}_{0}$, ${\rm{d}}F/F=\frac{F-{F}_{0}}{{F}_{0}}$. We chose a window that extended 5 s before and after saccade because at this value we retained a reasonable amount of data, while still finding patterns with time across the STAs (Fig. 3g). We excluded any cells that were recorded during an experiment that contained less than five saccades to the left and five saccades to the right (ten saccade-triggered responses total). Each of the saccades were required to be preceded and followed by a fixation that lasted at least 5 s. Of the 62,896 cells in the hindbrain that were identified as active by CaImAn, 36,527 cells had at least five left and right saccade-triggered responses that lasted 5 s or longer. Based on visual inspection of activity, we removed cells whose fluorescence activity near saccade was in the lowest 1% (375 total) of peak absolute STA values (peak absolute levels <14% d*F*/*F*) leaving 36,152 cells for eye-movement analysis. A 95% confidence intervals about the average (Fig. 2c–e) were found by resampling the saccade-triggered d*F*/*F* responses with replacement and calculating the STA for each resample (number of resamples = 100). We measured the lower and upper bounds of the confidence intervals as the 0.025 and 0.975 quantiles across the bootstrapped samples.

### Selection of eye-movement responsive cells

We ran a one-way ANOVA (MATLAB function *anova1*, Statistics and Machine Learning Toolbox) on saccade-triggered responses to test the null hypothesis that STA activity was equal at all time points versus the alternative that at least one time point had average activity that differed from the others. We considered a neuron as being eye-movement responsive if we rejected the null hypothesis for either one of that neuron’s two STAs (the STA triggered around saccades to the left or right). We used the Holm–Bonferroni method^69^ to correct for multiple comparisons. This procedure varied the significance level for each comparison by the formula $\frac{\alpha }{N-j+1}$, where $j$ was the index of the comparison after sorting *p* values from low to high, $\alpha$ was the desired family-wise error rate, and $N$ was the total number of comparisons. We set $\alpha$ to 0.01 and set the number of comparisons to 72,304 (36,152 active cells with STAs available for analysis times two to account for both saccade directions, see “Methods” section “Saccade-triggered average calculation”). We rejected the null hypothesis for 6,712 cells (19% of 36,152 active cells). The probability that a hindbrain cell is eye-movement responsive is therefore 0.05 (the probability that a given active hindbrain cell is eye-movement responsive, 0.19, times the probability that a hindbrain cell is active, 62,896/238,191).

### Principal component analysis of saccade-triggered averages

We ran a PCA to search for lower-dimensional representations of STAs across the population of eye-movement responsive cells. We combined STAs of deconvolved fluorescence from all eye-movement responsive cells (across all planes and fish recorded) and from both directions (around saccades to the left and right) resulting in a matrix, ${\bf{f}}$, that had $N=13\text{,}424$ rows (6,712 cells times two directions) and $T=31$ columns (time around the saccade is evaluated at 31 discrete-time bins of size 1/3 s). To focus our analysis on the variations in dynamics across cells, we divided each STA by its L2 norm before performing PCA,

1 $${f^{\prime} }_{{it}}=\frac{{f}_{{it}}}{\sqrt{\mathop{\sum }\limits_{a=1}^{31}{f}_{{ia}}^{2}}},$$

for $i=1,\ldots ,N$, $t=1,\ldots ,T.$ PCA (computed using MATLAB’s Statistics and Machine Learning Toolbox function *pca*) applied to ${\bf{f}}^{\prime}$ resulted in a matrix, ${\bf{u}}$, of 31 orthonormal basis vectors (each $T$ elements long) which we refer to as components (Fig. 3b) and a matrix of coefficients, ${\bf{c}}$ ($N$ × $T$ elements; Fig. 3c), that scale ${\bf{u}}$ such that,

2 $${f^{\prime} }_{{it}}-\frac{1}{N}\mathop{\sum }\limits_{j=1}^{N}{f^{\prime} }_{{jt}}=\mathop{\sum }\limits_{k=1}^{31}{c}_{{ik}}{u}_{{kt}},$$

for $$i=1,\ldots ,N$$, $$t=1,\ldots ,T.$$

To illustrate the main features of variation across the population of neurons recorded, we focused on the coefficients corresponding to the first three components after determining that these components explained the majority of the variance (Fig. 3a). In Fig. 3c, the coordinates ${c}_{i1}$, ${c}_{i2}$, and ${c}_{i3}$ are plotted on the ${c}_{1}$-axis, ${c}_{2}$-axis, and ${c}_{3}$-axis, respectively, for $i=1,\ldots ,N$. We normalized these coefficients to have unit norm,

3 $${c^{\prime} }_{{ij}}=\frac{{c}_{{ij}}}{\sqrt{\mathop{\sum }\nolimits_{a=1}^{3}{c}_{{ia}}^{2}}},$$

for $i=1,\ldots ,N$, $j=1,2,3$. We then transformed ${{\bf{c}}}^{\prime}$ into spherical coordinates (see Fig. 3c).

4 $$r=\sqrt{{c}_{i1}^{{\prime} 2}+{c}_{i2}^{{\prime} 2}+{c}_{i3}^{{\prime} 2}}=1,$$

5 $${\Theta }_{i}={{\arcsin }}\left({c^{\prime} }_{i3}\right)$$

6 $${\Phi }_{i}=\arctan \left(\frac{c^{{\prime} }_{i2}}{c^{{\prime} }_{i1}}\right),$$

for $i=1,\ldots ,N$. The elements ${\Phi }_{i}$ and ${\Theta }_{i}$ contain the ${i}{{\rm{th}}}$ STA’s values of the angles $\phi$ and $\theta$ shown in Fig. 3c and are used to construct Fig. 3c–h, and Supplementary Figs. 2 and 3. Note that we are working with latitude, which we have defined to be 0 at the equator in Fig. 3c, d, instead of the azimuthal angle ($90-\theta$) typically used when working in spherical coordinates. The principal advantage of working in spherical coordinates is that we can examine two-dimensional plots without sacrificing the temporal information stored in the first three principal components. The phases of ${\boldsymbol{\Phi }}$ and ${\boldsymbol{\Theta }}$ were chosen so that the ${i}{{\rm{th}}}$ STA whose temporal profile is equal to components one, two, or three would have coefficients, $\left({\Phi }_{i},{\Theta }_{i}\right)$, equal to (0°, 0°),(90°, 0°), (0°, 90°), respectively, as shown in Fig. 3c. The two-dimensional probability density function over ${\boldsymbol{\Phi }}$ and ${\boldsymbol{\Theta }}$ shown in Fig. 3d was measured using a normal Gaussian kernel smoothing function (MATLAB, Statistics and Machine Learning Toolbox function *ksdensity* with bandwidth parameter equal to 10° for ${\boldsymbol{\Phi }}$ and 3° for ${\boldsymbol{\Theta }}$).

Each population average shown in Fig. 3g is constructed by averaging together all non-normalized STAs triggered to saccades to the left with a specific value of $\phi$. For example, the average under the column $\phi$ = 105 was constructed by first finding the STAs triggered to saccades to the left with $\phi$ within 15° of 105 and then averaging these together. Letting ${{\bf{f}}}_{i,:}$ denote the ${i}{{\rm{th}}}$ STA and ${{\mathcal{S}}}_{105}$ denote the set of integers that index leftward STAs with $\phi$ = 105, ${{\mathcal{S}}}_{105}=\big\{i|{97.5\le \Phi }_{i} \,<\, 112.5,\text{and}{{\bf{f}}}_{{\boldsymbol{i}},}:\,\text{is}\,\, \text{an}\,\, \text{average}\,\, \text{of}\,\, \text{responses}\,\, \text{triggered}\,\, \text{to}\,\, \text{leftward} \,\,\text{saccades}\big\}$, the population average under the column $\phi$ = 105 is computed as

7 $$\frac{1}{{\rm{|}}{{\mathcal{S}}}_{105}{\rm{|}}}\mathop{\sum}\limits_{k\in {{\mathcal{S}}}_{105}}{{\bf{f}}}_{k,{\rm{:}}},$$

where $|{{\mathcal{S}}}_{105}|$ denotes the number of STAs indexed by the set ${{\mathcal{S}}}_{105}$. The population averages shown in Supplementary Fig. 3a are constructed in the same way except with the condition that STAs are triggered to saccades to the right. The population averages shown in blue in Fig. 3f are constructed in the same way except without the restriction on saccade direction. Note that the population averages shown in Fig. 3f, g and Supplementary Fig. 3a do not use the L2-normed responses, are constructed using all principal components and only use the angles in ${\boldsymbol{\Phi }}$ to group STAs. Therefore, the population averages that are displayed can reflect variations not captured by the first three components.

### *K*-means clustering of saccade-triggered averages

*K*-means was used to cluster the normalized coefficients (defined in Eq. (3) and associated text) found by PCA on STAs from eye-movement responsive cells (Supplementary Fig. 2). For each cell, we focused on the normalized coefficients that scale the first three principal components. We created a six-dimensional vector by combining the normalized coefficients that correspond to the STA triggered to saccades to the left and right. To choose the number of clusters, we ran nine *K*-means analyses, using a different number of clusters on each run (between 2 and 10) to group the combined coefficients, and we used the silhouette value to measure cluster quality. The silhouette value for an individual vector measures how close that vector is to other vectors in its own cluster relative to its distance with vectors in other clusters. The value for the ${i}{{\rm{th}}}$ vector is defined as the minimum average distance from the ${i}{{\rm{th}}}$ vector to all vectors in different clusters than the ${i}{{\rm{th}}}$ vector, *b*_*i*_, minus the average distance from the ${i}{{\rm{th}}}$ vector to other vectors in the same cluster, *a*_*i*_. The silhouette value is normalized by max*(a*_*i*_ *, b*_*i*_*)* in order for it to range from +1 to −1 with vectors that are well matched to their cluster having values near +1, and with vectors that are randomly clustered having values near 0.

### Anatomical projections

We created maps of SR cell locations (Fig. 6d, e) and of the angle $\varphi$ shown in Fig. 3c (computed as described in Eq. (6) and related text) from STAs triggered to saccades to the left (Fig. 3h)/right (Supplementary Fig. 3b) for each fish and imaging plane. Since some regions were imaged with more fish than others (Supplementary Fig. 1c), these maps were created using a randomly selected subset of the eye-movement responsive cells. To adjust for variation in the number of fish sampled across brain regions (all regions were sampled by at least three fish), we assigned each cell a probability of being included in the map equal to three divided by the number of fish sampled at that cell’s location. Each projection was made by first registering cell locations to the Z-Brain (see “Methods” section “Registration of individual planes to the Z-Brain atlas”) and then binning cells into 2D square bins (5 µm in length) appropriate for the given projection. The modes shown in Fig. 3h and Supplementary Fig. 3b were computed by first constructing a histogram (15° bins) of the values of $\varphi$ from STAs triggered to the left/right from cells registered to a given square in the projection. The color displayed was determined by the value where the histogram peaked or, in cases where the histogram had multiple modes, was determined by the value at a randomly chosen peak.

### Selection of SR cells

An eye-movement responsive cell was classified as an SR cell if its d*F*/*F* response before upcoming saccades was significantly correlated with time before saccade. We measured two Spearman correlations for each eye-movement responsive cell. One correlation was computed (MATLAB, Statistics and Machine Learning Toolbox function *corr* with option *type* set to Spearman) on values of d*F*/*F* and time before saccade concatenated from all fixations before saccades to the left. The other correlation was computed on values of d*F*/*F* and time before saccade concatenated from all fixations before saccades to the right. The Spearman correlation can be used to measure monotonic (not only linear) relationships between two variables $X$ and $Y$. It is calculated as the standard Pearson correlation coefficient applied to the ranks of $X$ and $Y$. We did not interpolate fluorescence activity before computing the correlation coefficients. Since we were interested in cells whose activity is related to upcoming saccade, we did not include activity that was within 2 s of the previous saccade, where eye-movement responsive cells might have post-saccadic fluorescence decays. We computed a *p* value for each correlation by testing the hypothesis that rho = 0 against the alternative that the correlation was greater than 0 (*tail* option set to *right*). A neuron was considered to have significant pre-saccadic activity if we rejected the null hypothesis for any of the cell’s two correlation coefficients at a significance level of 0.01. To correct for multiple comparisons, we used the Holm–Bonferroni method (as described in “Methods” section “Selection of eye-movement responsive cells”) with the number of comparisons set to 13,424 (the number of eye-movement responsive cells times two directions).

### Measuring SR cell activity rise time

We measured the time when an SR cell’s activity rose above baseline by determining when its deconvolved fluorescence crossed a threshold near zero (0.1). Based on the visual inspection of deconvolved SR traces, we found that the nonnegativity constraint used to compute deconvolved fluorescence facilitated the distinction between times when the cell was responsive from times when it was not (Fig. 4a). Epochs of time where the deconvolved estimate was equal to, or nearly equal to, zero were interpreted as epochs where the cell was not responsive. Therefore, we measured SR cell activity rise time for each fixation (Fig. 4b), as the time point before its deconvolved fluorescence increased >0.1.

### Measuring rate of pre-saccadic rise in single SR cells

We estimated the rate of pre-saccadic rise in SR cells by finding the slope of the best-fit line of pre-saccadic deconvolved fluorescence with time. To construct the best-fit line we used time from pre-saccadic rise to the time of upcoming saccade as a regressor to a linear regression that fit deconvolved fluorescence values. The linear approximation was reasonable for 72% of the fixations (correlation between regression fit and data was >0.4). We excluded fixations where we were unable to measure the slope with linear regression.

### Predicting saccade direction using choice probability

We predicted saccade direction using interpolated SR activity before saccadic events. Throughout this section, the phrase “interpolated SR activity” refers to linear interpolation of deconvolved fluorescence activity to a grid of equally spaced time points (using 1/3 s bins) starting from the previous saccade to the upcoming saccade.

The CP is a commonly used metric in neurophysiology and psychophysics experiments that quantifies how well an ideal observer can predict animal behavior^37^. In a typical neurophysiology application, the CP measures relationships between neuronal discharges and binary behavioral choices. We adapted this metric by using the spontaneous decision to saccade to the left or the right as our binary behavioral variable, and population average deconvolved fluorescence at a given time before saccade as our neural read-out. At discrete time points before upcoming saccades, we made two histograms of interpolated SR activity. One distribution, referred to as the noise distribution, was comprised of values of interpolated SR activity before saccades to the right (left) from SR cells significantly correlated with upcoming saccades to the left (right). The other distribution, referred to as the signal distribution, was comprised of interpolated SR activity before saccades to the left (right) from SR cells significantly correlated with upcoming saccades to the left (right). Saccade direction was predicted using a threshold on population activity. We plotted the fraction of interpolated SR activity from the signal distribution that was above threshold (the true positive rate) versus the fraction of interpolated SR activity from the noise distribution that was above threshold (the false positive rate) across multiple threshold values. The CP was calculated as the area under the resulting curve, which would equal 0.5 if activity and upcoming saccade direction were not related. To estimate the variability in CP, we computed multiple CPs each conditioned on a different fixation duration (fixing durations to values 2–20 s) and then computed the CP SEM across fixation durations.

### Predicting saccade times using a ramp-to-threshold model

We predicted when a saccade would occur by calculating a running estimate of population activity slope and then plugging this estimate into a ramp-to-threshold model to predict when population activity will cross a threshold, $\kappa$. We first measured $\kappa$ by averaging deconvolved fluorescence reached at the time of saccade across all SR cells in our dataset. For each fixation duration, we then calculated population average activity before upcoming saccade by combining (across all cells and fish) interpolated SR activity (Fig. 5e; we used eighteen nonoverlapping values of fixation duration from 3.5 ± 0.5 to 20.5 ± 0.5 s). We modeled the population average dynamics, $y(t)$, at values of time $t$ after population activity begins to rise, with the following linear ramp equation

8 $$y={Dt},$$

where $D$ is the population activity slope. After a time, ${t}_{r}$, a saccade will occur and $y$ should equal $\kappa$ if the ramp-to-threshold model is accurate, e.g.,

9 $$y\left({t}_{r}\right)=\kappa =D{t}_{r}.$$

We constructed a running estimate of $D$ by first measuring when the actual population activity, $\widetilde{y}\left(t\right),$ began to rise. Note that we are distinguishing the population activity measured from data, $\widetilde{y}\left(t\right)$, from the model of population activity, $y(t)$, specified by Eq. (8). We measured when $\widetilde{y}\left(t\right)$ began to rise as the time when the derivative in $\widetilde{y}\left(t\right)$ crossed a threshold of 35 (arbitrary units). Note that under our convention we set this event to occur at time 0. The derivative was approximated as the difference between population activity at each time point divided by the interpolated time bin interval, $\triangle t$, of 1/3 s. We created a running estimate of $D$ using the median value of the derivative from time 0 until time, $t$,

10 $$\widetilde{D(t)}={\rm{median}}\left({\left\{\frac{\widetilde{y}\left({t}^{{\prime} }+\triangle t\right)-\widetilde{y}({t}^{{\prime} })}{\triangle t}\right\}}_{0}^{{t}^{{\prime} }=t}\right).$$

We substituted our running estimate of the slope into Eq. (9) to yield a running estimate of ${t}_{r}$

11 $$\widetilde{{t}_{r}(t)}=\frac{\kappa }{\widetilde{D(t)}},$$

where we have used the ~ symbol to denote estimates of model parameters. Using our estimate $\widetilde{{t}_{r}(t)}$, we could predict the amount of time until upcoming saccade for any given value of $t$, as $\widetilde{{t}_{r}(t)}-t$. Figure 5h shows predictions of the time until upcoming saccade made by varying the value of *t* from one time point after population activity begins to rise to one time point before saccade for each fixation duration.

In the text and in Supplementary Fig. 6, we also present results showing how well the population average activity is approximated by a ramp-to-threshold model. In these cases, we did not create a running estimate of the slope, but instead only created one slope estimate by setting ${t=t}_{r}$ in Eq. (10). We used all cells to estimate $\widetilde{D\left(t\right)}$ when we forecasted saccade times for Fig. 5h, i; however, using all cells would have led to overfitting in Supplementary Fig. 6. To prevent overfitting in this case, we only used a random subset of all cells (60%) to measure $\widetilde{y}\left(t\right)$. We used this measurement to compute $\widetilde{D}$ (via Eq. (10) with ${t=t}_{r}$) and then plugged $\widetilde{D}$ into Eq. (8) to predict population activity. We then tested this prediction against a new measurement of population activity constructed from the remaining 40% of cells. To determine model accuracy, we repeated this procedure on 10,000 randomly selected subsets from randomly chosen fixation durations.

### Saccade time predictions by an ideal observer

As a control, we determined the fraction of saccades that could be accurately predicted given knowledge of the elapsed time since last saccade and the distribution of fixation durations. Given this information, an ideal observer could predict upcoming saccade times by guessing a time that minimizes some cost function that measures error between the actual saccade time and the guessed time. We tried three cost functions (mean-squared error, mean-absolute deviation, and all-or-none error) and report results in the text from the one that performed the best (all-or-none error).

### Cluster laser ablations

We targeted regions along the dorsal–ventral axis that were 30 µm dorsal of the medial longitudinal fasciculus since this is where we found most eye-movement responsive cells. Ablations were performed with the same microscope used for imaging. Cluster ablations^40^ were created by focusing the laser to an area smaller than a single cell and then increasing the average laser power to values between 130 and 150 mW for 1–5 s. We repeated this procedure until we saw a lesion, which we determined by looking for a multi-spectrum spot that was much brighter than the fluorescence of surrounding tissue. Lesion sizes with this procedure were generally ~5 µm in diameter. To increase the size of the lesion we lowered the average laser power to values of 30–50 mW and scanned the ablated region at these lower powers, which caused the lesion size to grow. We stopped scanning the ablated region once it grew to ~30 µm in diameter. We waited between 30 and 120 min after ablation before recording post-lesion eye movements.

To estimate the fraction of SR cells lesioned in a given fish during cluster ablations (Fig. 7d and text), we registered the ablated animals and SR cell locations to the bridge brain (see “Methods” section “Registration of individual planes to the Z-Brain atlas”) and then calculated the number, ${n}_{c}$, of SR cells (locations shown in Fig. 6) that fell within a cylinder (30 µm radius, 60 µm side length along dorsal–ventral axis) centered about the location of peak ablation damage. To determine the cylinder center, we manually inspected time-averaged images of each the ablated region (Fig. 7a) to find planes containing a bright, multi-spectrum fluorescence characterizing ablation damage. We found the plane with the maximal damage, selected similar points between this plane and the bridge brain (as demonstrated in Supplementary Fig. 1a) and then created a 2D affine matrix to transform the center of the registered lesion to the bridge brain. The fraction ablated was equal to ${n}_{c}$ divided by the total number of SR cells used to construct the map.

### Single-cell laser ablations

In a separate set of experiments, we targeted individual SR neurons for ablation. We imaged neuronal activity in several planes (185 µm^2^ in size) centered on regions most densely populated with SR cells (dorsal of the Mauthner cell between rhombomeres 2 and 6, see Fig. 6). During the experiment, we analyzed saccade-triggered activity of all cells in the imaging planes to identify the locations of eye-movement responsive cells (using the ANOVA based procedure described in “Methods” section “Selection of eye-movement responsive cells”) and then identified which of these cells were SR cells (using the procedure described in “Methods” section “Selection of SR cells”). The total time spent imaging and processing activity before ablation varied between 10 min and 2 h. After processing, we targeted four to seven randomly chosen SR cells for ablation. Before each ablation, we manually corrected for any changes in cell-center location that might have arisen during SR cell identification. Manual correction was performed by zooming into a region containing a cell of interest, taking a new time-averaged image, and reidentifying the SR neuron location by comparing the new with the original time-averaged image where the SR cell was identified. We did not attempt an ablation if the experimenter could not reidentify the cell of interest after processing. We ablated individual neurons by focusing a high-powered, pulsed femtosecond laser (810 nm, 400–500 mW after the objective) on the center of SR cells for a brief (2–5 ms) period of time^28,29,70^. This procedure resulted in the loss of one to three cells per ablation attempt (Fig. 7b) at the depths where SR cells were targeted (40–70 µm below the surfaced). In some cases, ablations did not occur even after three to five attempts, most likely due to laser power absorption from pigmentation. We did not try to ablate a cell after attempts. In one experiment, we repeated three cycles of finding cells of interest in a single plane, ablating these cells, then searching for more cells of interest in subsequent planes. In all other experiments we imaged, identified cells of interest, and then ablated these cells. At the end of the experiment, we took time-averaged images of the entire hindbrain dorsal of the Mauthner cell and used this stack to register ablated cells to the Z-Brain Atlas. For control cell ablations, we performed the same procedure but targeted cells that failed to pass our criteria for being considered eye-movement responsive.

### Analysis of behavior following laser ablations

For each fish, we measured fractional changes in median fixation duration after ablation. Since we were concerned with a spontaneous behavior, we could not control the number of fixations that were recorded during the timeframe of each experiment. As a result, our measurements would have had different accuracies per animal (Supplementary Table 1), if we calculated fractional changes without accounting for the different number of samples per fish. To control for this difference in accuracy, while using all our data, we made repeated measurements of the fractional change per fish with each repeated measurement computed using the same number of fixations before and after ablation. For each repeated measurement, we randomly sampled without replacement ${N}_{{{\min }}}$ fixations before and after ablation. The exact value of ${N}_{{{\min }}}$ was based on animals with the fewest number of fixations available after excluding animals that stopped making saccades after ablation; animals whose average saccade rate never increased above one saccade per direction per minute were excluded (*n* = 3 animals from cluster ablation experiments, 2 from treated group, and 1 from control). Specifically, if we denote the number of fixations before or after ablation (indexed by $i$) from animal $j$, as ${n}_{{ij}}$, then ${N}_{{{\min }}}=\mathop{{{\min }}}\nolimits_{i,j}{n}_{{ij}}$. The number of times we repeated each measurement of fractional change in median fixation duration varied per fish and was determined by how many more fixations each animal made compared to ${N}_{{{\min }}}$. Specifically, the number of times we repeated each measurement for animal $j$ equaled ${\rm{round}}\,(\frac{\mathop{{{\min }}}\nolimits_{i}{n}_{{ij}}}{{N}_{{{\min }}}})$. The fractional change in median fixation duration was computed as the difference in median fixation duration (after minus before ablation) divided by the median fixation duration before ablation. Using $l$ to denote the index of the repeated measurement in animal $j$ and ${t}_{{ijml}}$ to denote the ${m}{{\mathrm{th}}}$ randomly sampled fixation duration (integer ${m}$ varies from 1 to ${N}_{{\rm{min }}}$) in condition $i$ ($i$*=*1 denotes before ablation and $i$ = 2 denotes after ablation), the fractional change in fixation duration was computed as:

12 $${y}_{{jl}}=\frac{\mathop{{\rm{median}}}\nolimits_{m}{t}_{2{jml}}-\mathop{{\rm{median}}}\nolimits_{m}{t}_{1{jml}}}{\mathop{{\rm{median}}}\nolimits_{m}{t}_{1{jml}}},$$

for $l$ = 1, 2, …, ${\rm{round}}(\frac{\mathop{{{\min }}}\nolimits_{i}{n}_{{ij}}}{{N}_{{{\min }}}})$ and *j* = 1, 2, …, total number of animals.

To determine how the variability in cluster ablation results depended on the sample size used to compute the change in fixation duration, ${N}_{{{\min }}}$, we reran the entire procedure described >13 times each time setting a different floor to ${N}_{{{\min }}}$ (floor values equaled 55, 65, 75, …175). For each floor value, ${N}_{{{\min }}}$ was computed after removing every animal whose minimum value of fixations either before or after ablation was below the floor value (see minimum number of fixations per fish in Supplementary Table 2). At each floor value, we computed the Pearson correlation coefficient between the elements of ${\bf{y}}$ and the fraction of cells removed during a cluster ablation (see “Methods” section “Cluster laser ablations” for a description of how we estimated the fraction of SR cells ablated for each animal). Since the elements of ${\bf{y}}$ depend on the random samples chosen, we repeated our sampling procedure 100 times for each floor value to obtain 100 samples of ${\bf{y}}$, and subsequently 100 correlation coefficients and then computed the median across these samples resulting in 13 median correlation coefficients. In the “Results” section regarding cluster ablations (see “Focal laser ablations identify SR cells as indispensable for setting spontaneous fixation durations”), we presented the average correlation across these 13 values (${N}_{{{\min }}}$ varied between 57 and 176 fixations, *n* varied between 10 and 29 animals, see Supplementary Table 2 to determine exact *n* at a given floor). At each floor value, we also ran a one-sided, two-sample KS test between the 100 samples of correlation coefficients and 100 coefficients obtained by correlating randomly shuffled elements of ${\bf{y}}$, with the fraction of ablated SR cells. This resulted in 13 *p* values since we used 13 floor values. All 13 *p* values were significant at a criterion of 0.001 using the Holm–Bonferroni method to control for multiple comparisons. As expected, the variability in correlation coefficients increased when too few samples, ${N}_{{{\min }}},$ were used to compute each fractional change; the average correlation across the eight values with the largest values of ${N}_{{{\min }}}$ (${N}_{{{\min }}}$ varied between 105 and 176 fixations, *n* varied between 10 and 20 animals) = 0.33. The values plotted in Fig. 7d are from one of the hundred samples of ${\bf{y}}$ at ${N}_{{{\min }}}$ = 57 that contained a typical correlation coefficient; Supplementary Fig. 8d shows results from the same run used to construct Fig. 7d.

In the “Results” section regarding single-cell targeted ablations, we repeated our sampling procedure 100 times to calculate 100 samples of the mean of ${\bf{y}}$ and presented the minimum, maximum, and median across these samples. We did not remove animals for single-cell ablation analysis (${N}_{{{\min }}}$ = 33 fixations; see Supplementary Table 3 for the number of repeated measurements per animal $j$, i.e., ${\rm{round}}(\frac{\mathop{{{\min }}}\nolimits_{i}{n}_{{ij}}}{{N}_{{{\min }}}})$, where the conditions before and after ablation are indexed by $i$). Figure 7e shows one of the hundred runs of ${\bf{y}}$ that results in a typical change between SR and control-targeted groups. The sham ablation results presented in Fig. 7e were computed without making repeated measurements using ${N}_{{{\min }}}$ = 33 fixations per animal to compute fractional change in median fixation duration. For each sample of ${\bf{y}}$ for control and SR-targeted animals, we ran a Wilcoxon rank-sum test (100 tests in total) of the null hypothesis that the medians are equal between the distribution of ${\bf{y}}$ for SR-targeted and the distribution of ${\bf{y}}$ from control animals against the alternative that the median is greater in SR-targeted animals. The minimum, maximum, and median *p* values across the 100 runs were also presented in the results. MATLAB function *ranksum* with the appropriate value of *tail* was used to perform the one-sided Wilcoxon tests.

### Reporting summary

Further information on research design is available in the Nature Research Reporting Summary linked to this article.

## Supplementary information

Supplementary Information

Reporting Summary

## Data Availability

The calcium and eye movement traces that support the findings of this study are available in figshare with the identifier 10.6084/m9.figshare.14558064.v1 (ref. ^71^).  Source data are provided with this paper.

## Source data

Source Data

## Code availability

The code used for data analysis in this study can be downloaded from GitHub at https://github.com/alxdroR/SRNatComm21.git [10.5281/zenodo.4743159]^72^.
