# Supplementary material for: Ramp-to-threshold dynamics in a hindbrain population controls the timing of spontaneous saccades
Source: Nat Commun. 2021 Jul 6;12:4145. doi: 10.1038/s41467-021-24336-w (PMC8260785; doi:10.1038/s41467-021-24336-w)
Supplement: Supplementary file 1 — Supplementary Information [file 41467_2021_24336_MOESM1_ESM.pdf]

Supplementary Information for
Ramp-to-Threshold Dynamics in a Hindbrain Population Controls the Timing of Spontaneous Saccades

Alexandro D. Ramirez[1], Emre R.F. Aksay[1]

[1] Department of Physiology and Biophysics; Weill Cornell Medicine; New York; New York; 10065*email: alr2038@med.cornell.edu

Supplementary Table 1. Increase in median fixation duration along with 95% confidence intervals (in brackets) following single-cell ablations without controlling for differences in number of fixations per animal. Notice how the variability changes per fish. Values are reported in seconds. Confidence intervals were found by bootstrapping with 10,000 samples. Note that animals with SR-targeted ablations are different than animals with control ablations.

SR Ablations	Control Ablations
-0.4, [-2, 1]	-3, [-6, -1]
1.5, [-2, 4]	-1, [-4, 2]
2.3, [1, 4]	-0.2, [-2, 2]
3.4, [-2, 5]	-0.2, [-2, 2]
5.8, [4, 7]	3.7, [0, 10]
8.2, [5, 12]	5.6, [4, 7]
8.4, [5, 10]	6.3, [3, 9]
9, [5, 14]	6.4, [3, 10]
14.3, [13, 18]	12.4, [10, 16]
22.7, [16, 29]	22.3, [18, 28]

Supplementary Table 2. Minimum number of fixations before or after ablation per fish using cluster ablation data (Fig. 7d, Supplementary Fig. 8d and associated text). Note that the animals with spinal cord ablations are different than the animals with cluster hindbrain ablations

Hindbrain Ablations	26	45	52	57	59	63	70	105	123	126	132	149	160	172	173	176	187	223	226	249	269	277	312	319	351
Spinal Cord	54	77	88	93	95	104	117	142																	

Supplementary Table 3. Number of times we measured fractional change in median fixation duration per fish using single-cell ablation data (Fig. 7e and associated text). Note that animals with SR-targeted ablations are different than animals with control ablations.

SR Ablations	4	3	2	4	8	2	3	3	10	5
Control Ablations	3	1	1	5	5	5	7	4	6	3

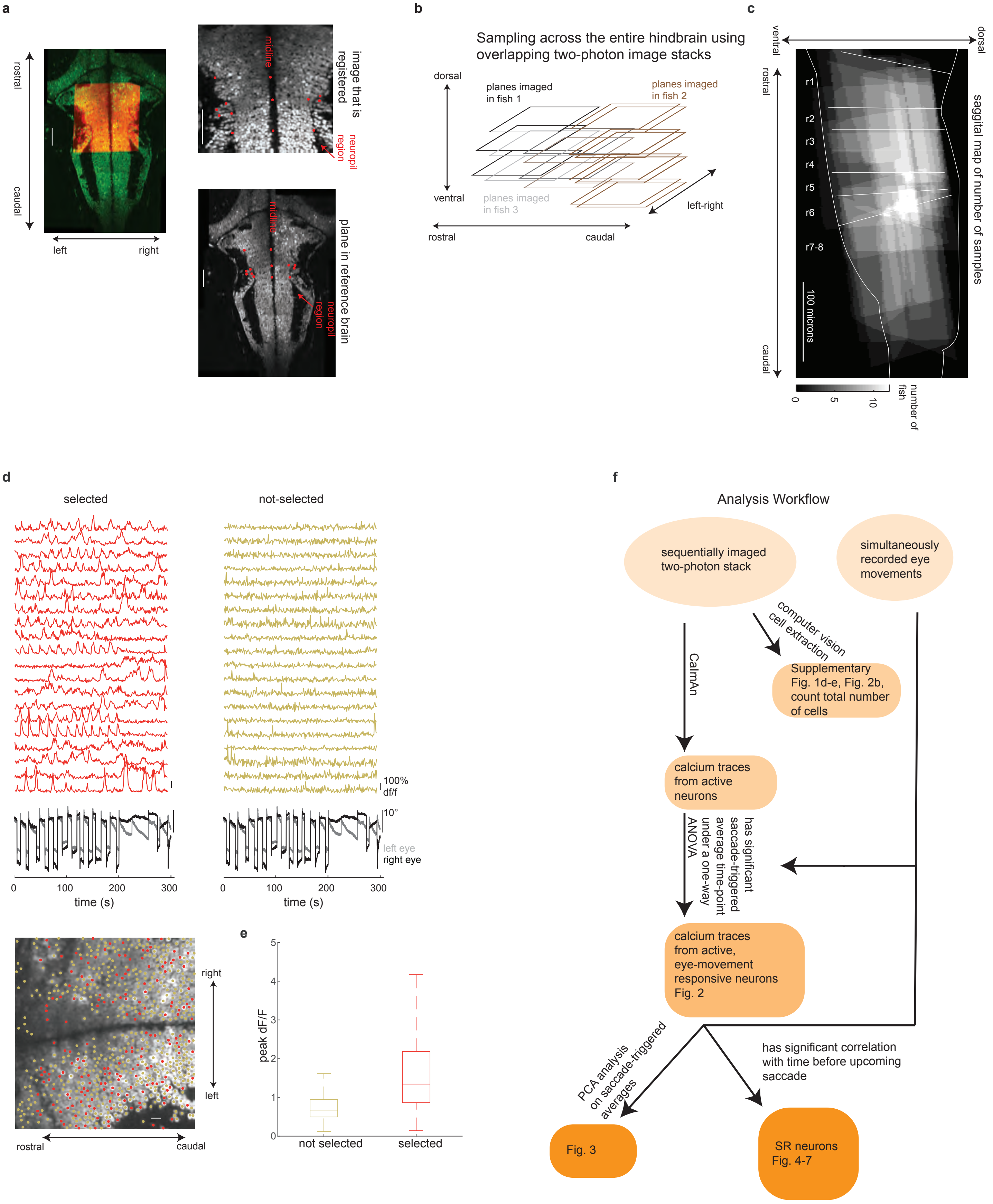

Supplementary Figure 1

Supplementary Figure 1. Methods used for imaging and analyzing cells across the hindbrain. (a) Demonstrating the procedure used for registration. Time-averaged calcium image from reference brain shown in green and overlapping image that is registered in orange. Similar points (red dots) were found between registered images and the reference brain using corresponding features such as the midline location and neuropil boundaries. These points were used to compute a transformation matrix for registration (see Methods). Scale bar length is 50 microns. (b) Cartoon schematic of sampling method. Each square represents a horizontal imaging plane in the hindbrain. Overlapping stacks of horizontal planes (185x185 microns) in the hindbrain were imaged in multiple fish (different colors) and then registered to a reference brain resulting in hindbrain-wide imaging across the population. (c) Activity in each brain region was sampled across multiple fish. Sagittal projection from the hindbrain (grayscale values display the number of fish used to sample each voxel, n=20 fish in total). (d) Simultaneously recorded traces of dF/F along with eye movements demonstrating that neurons identified by the CaImAn algorithm (red) show greater fluctuations in fluorescence than cells classified as background by the algorithm (yellow; background cells localized using intensity, see Methods). Cell centers shown by color-coded dots in time-average image of h2b-GCaMP6f at the bottom. Scale bar length is 10 microns. Median peak dF/F of selected and non-selected cells displayed is 1.9 and 0.99 respectively. Similar results were seen across 422 planes collected from 20 fish, see (e). (e) Boxplots of maximum dF/F value across time from the population of cells selected by the CaImAn algorithm (red) and cells classified as background (yellow). Central line shows the median, box limits show the 25th and 75th percentiles, whiskers show 1.5 x interquartile range about upper and lower quartiles. We rejected the null hypothesis that the average peak dF/F values are equal for the two populations ($p<0.0001$, one-way ANOVA; $F=56,369$; n=196,494 non-selected cells, n=62,896 selected cells). Source data are provided in a Source Data file. (f) Analysis workflow detailing the steps used to derive cells examined in the manuscript.

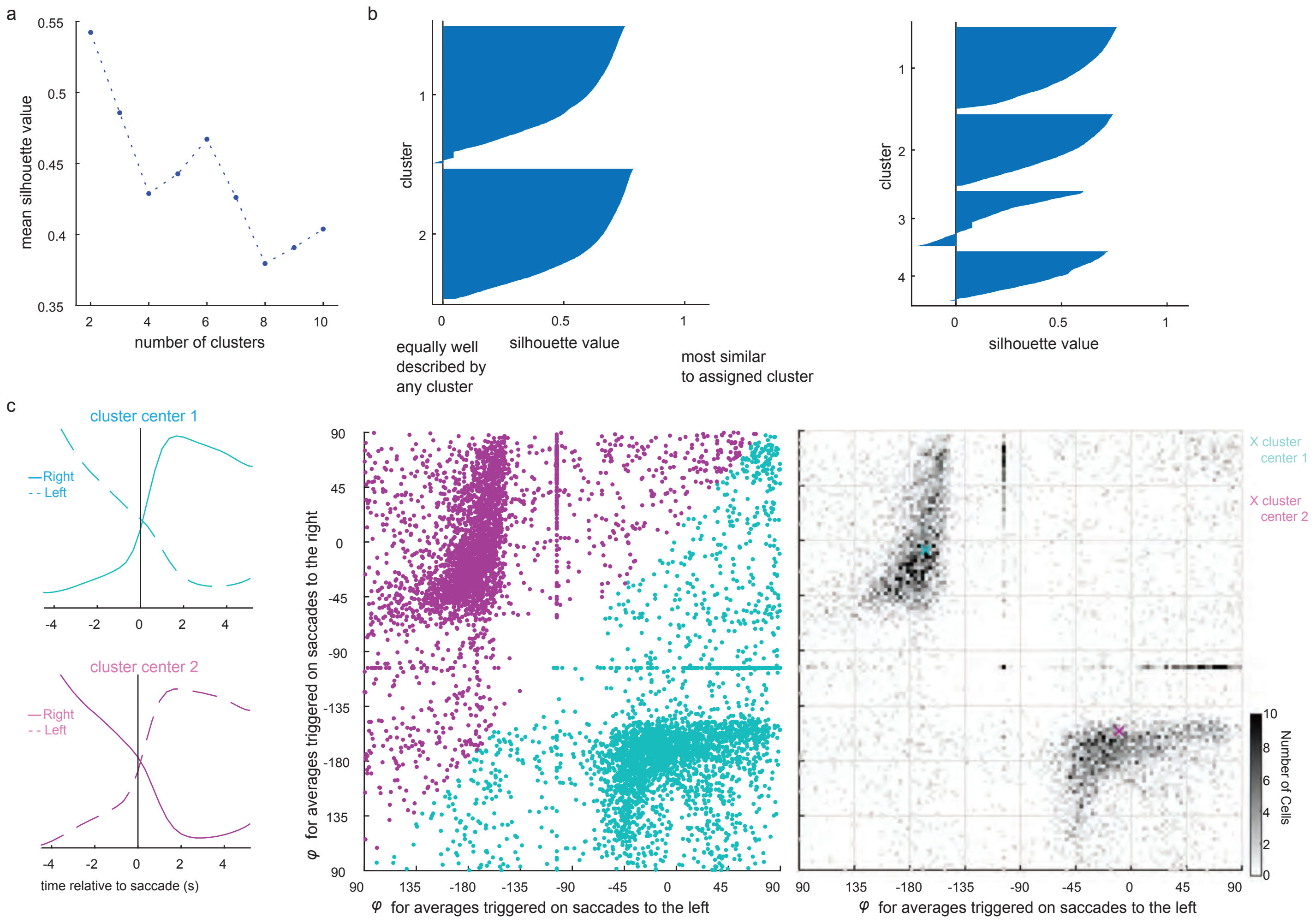

Supplementary Figure 2. K-Means Analysis of combined left and right saccade-triggered averages. (a) Mean silhouette value versus number of clusters. The largest average silhouette value (best clustering) is achieved using 2 clusters. (b) Silhouette plot showing distribution of silhouette values for all vectors after running K-means using two clusters (left panel) and four clusters (right panel). (c) (left panel) STAs around saccades to the left (dashed) and right (solid) corresponding to the cluster means found by K-means analysis using two clusters. The STAs displayed are created by linearly combining the first three components weighted by the coefficients corresponding to the first (top) or second (bottom) cluster center. The center of cluster 1 corresponds to STA pairs that increase in value following saccades to the right and decrease in value following saccades to the left. The center of cluster 2 shows similar activity but with the opposite directional preference. (middle panel) Scatter plot of angular coefficients (see Fig. 3c and accompanying methods), characterizing the STA around saccades to the left versus the coefficient characterizing the same cell's STA around saccades to the right. Each dot is colored according to its assigned cluster after running a K-means analysis on the plot with K=2. Each dot is from a different cell. (right panel) Two-dimensional histogram of coefficients plotted in the middle panel (bin size equals 1 degree). Source data are provided in a Source Data file.

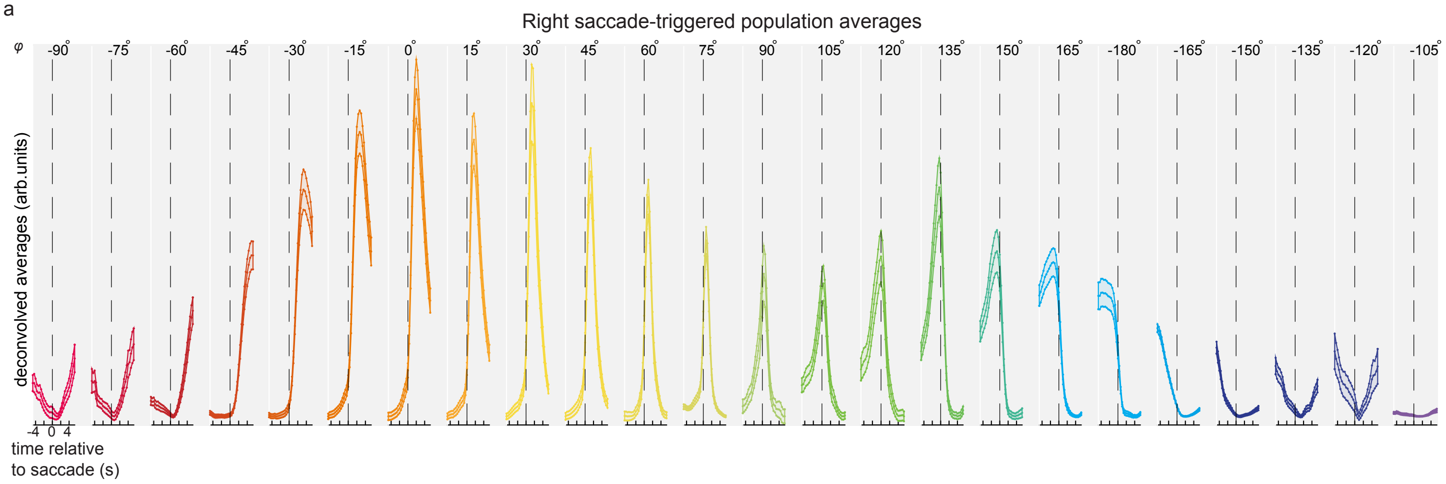

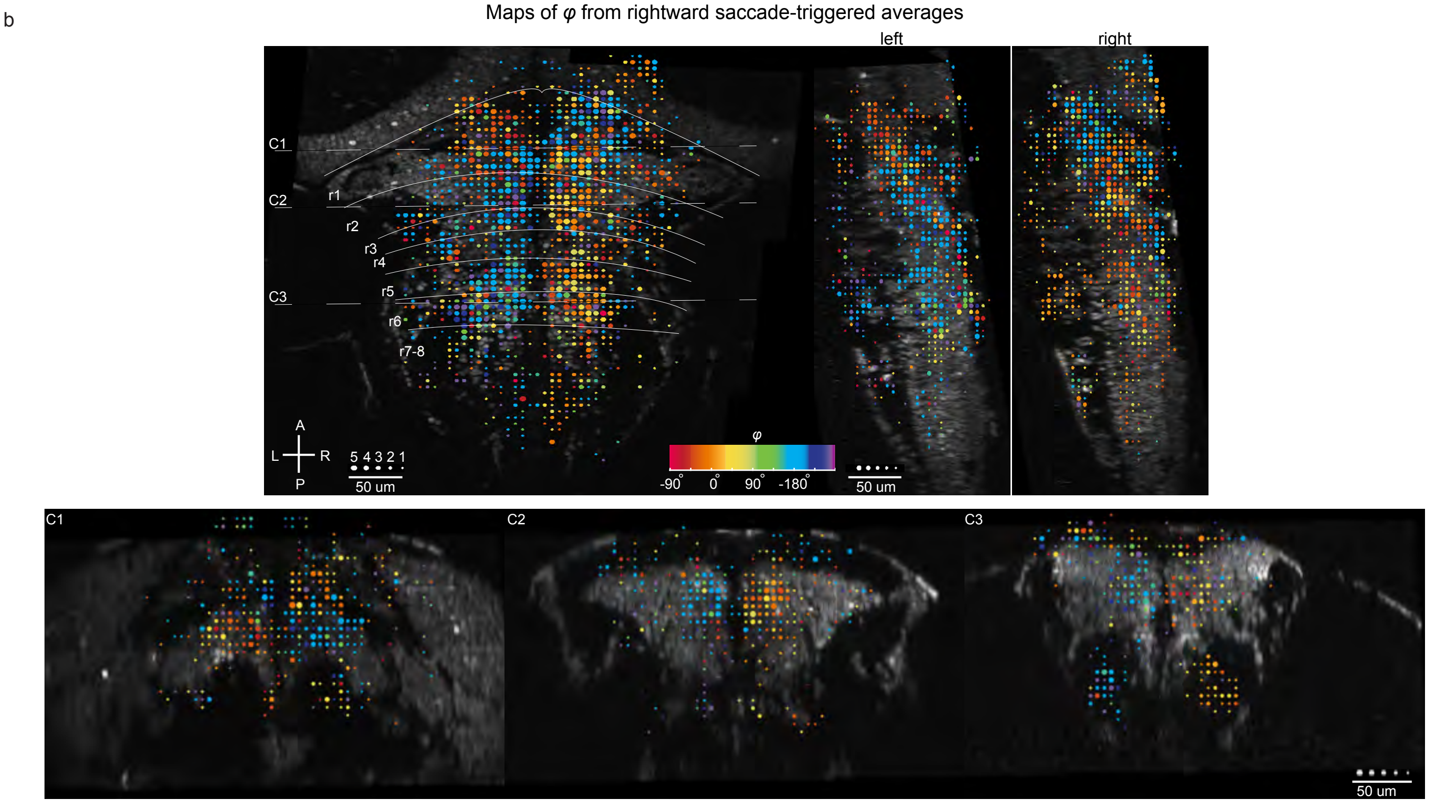

Supplementary Figure 3

Supplementary Figure 3. Functional and spatial distribution of eye-movement responsive activity triggered to saccades to the right. (a) Series of population-averaged STAs triggered to saccades to the right with φ equal to the value in the top row (within 15 degrees; see Fig. 3c and Methods section Principal Component Analysis for the definition of φ). Data shown as mean (solid line) +- SEM (shaded region). Varying number of cells: $\varphi =$ -90 (n=62 cells, 14 fish), -75 (n=68, 14), -60 (n=220, 17), -45 (n=405, 16), -30 (n=483, 16), -15 (n=421, 17), 0 (n=322, 16), 15 (n=282, 16), 30 (n=269, 18), 45 (n=254, 17), 60 (n=302, 17), 75 (n=303, 16), 90 (n=102, 16), 105 (n=98, 13), 120 (n=130, 16), 147 (n=135, 15), 150 (n=219, 16), 165 (n=353, 17), 180 (n=239, 16), -165 (n=787, 16), -150 (n=380, 17), -135 (n=72, 12), -120 (n=57, 15), -105 (n=412, 17). (b) Horizontal, sagittal and caudal projections of a sample of cells used to construct (a) (n=3,012 total cells examined over 18 fish). Color indicates most probable value of φ for cells within 5 micron bins (using the same color scheme as (a)). Circle size indicates number of cells within bin (largest is ≥5 cells). Coronal projections are made within 30 microns of the dashed lines marked C1, C2, and C3 in the horizontal map. r, rhombomere; L-R, left-right; A-P, anterior-posterior. Source data are provided in a Source Data file.

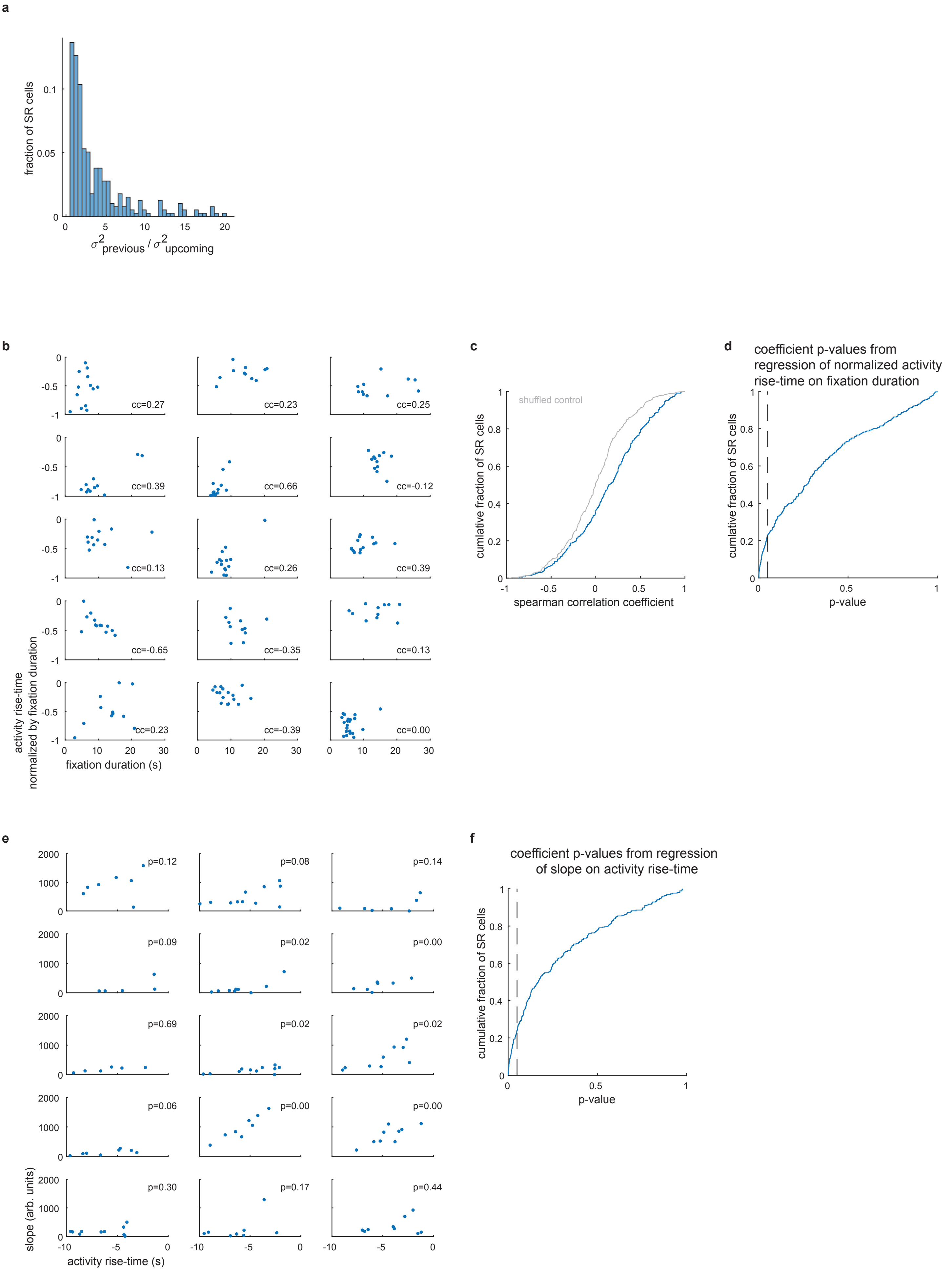

Supplementary Figure 4

Supplementary Figure 4. Single SR cell analysis of pre-saccadic rise features. (a) Histogram of the ratio of variances in activity rise-time (median ratio = 1.9; n=375 cells). $\sigma^2_{previous}$ [$\sigma^2_{upcoming}$] is the variance across fixations in the activity rise-time relative to the previous [upcoming] saccade. (b) Scatter plots of activity rise-time, relative to upcoming saccade, normalized by fixation duration versus fixation duration for 15 example cells. Each plot corresponds to a different cell. cc is the Spearman correlation coefficient between the plotted statistics. (c) Cumulative distribution of the Spearman cc between the variables plotted in (b) (blue line). Gray line shows statistics from the same cells after randomly shuffling the normalized time of rise. We rejected the null hypothesis that cc values from SR cells and shuffled controls come from the same distribution ($p<0.001$, n=372 cells, two-sample KS test). (d) Cumulative distribution of p-values for t-statistics of the two-sided hypothesis test that the regression coefficient from a linear regression run on the variables plotted in (b) equals zero or not. Only 23% percent of cells had a p-value less than 0.05 (dashed line). Amongst cells with $p<0.05$, 86 percent had the same trend (positive slope) as the population shown in Fig. 4g. (e) Scatter plots of rates of deconvolved fluorescence increase versus activity rise-time for 15 example cells. Each plot corresponds to a different cell. The number in the upper right corner of each plot reports the p-value for the *t* statistic of the two-sided hypothesis test that the regression coefficient from a linear regression run on the plotted variables equals zero or not. (f) Cumulative distribution of the p-values described in (e) (n=372 cells). 24 percent of the cells in the plot have $p<0.05$ (dashed line) and have the same trend (positive slope) as the population. All cells analyzed in this figure were required to be recorded along with at least 5 simultaneously recorded saccades. Cells were required to have either fixation durations (c)(d) or rise-times (f) of sufficient variability (interquartile range was greater than or equal to 1 second). Source data are provided in a Source Data file.

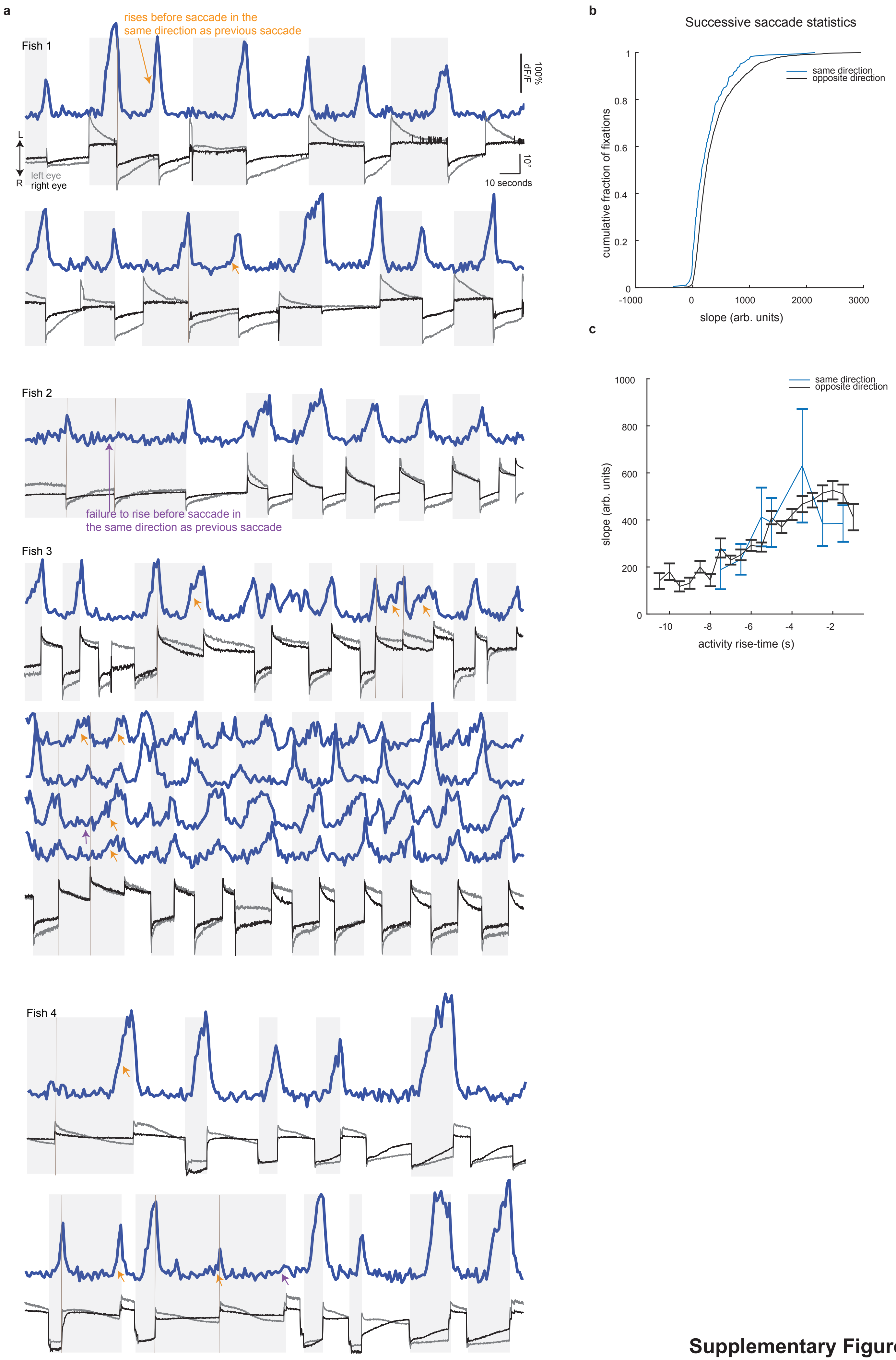

Supplementary Figure 5

Supplementary Figure 5. SR neuron activity conditioned on saccade patterning. (a) Simultaneously recorded eye position (black and gray traces) and single-cell dF/F responses (blue) from 10 SR cells (found in 4 different fish) recorded when the eyes made at least one saccade in the same direction as the previous saccade. Leftward/rightward directed saccades are made toward the direction labelled L/R. We highlight examples where SR neurons rise before saccades in the same direction as the previous saccade in orange. We also show cases where SR neurons failed to rise before saccades in the same direction as the previous saccade in purple. (b) Cumulative histogram of rates of pre-saccadic deconvolved fluorescence increase before a saccade in the same direction as the previous saccade (blue) and before a saccade in the opposite direction as the previous saccade (black). (c) Average slope of pre-saccadic deconvolved fluorescence increase as a function of pre-saccadic activity rise-time measured with respect to upcoming saccade before saccades made in the same (blue)/opposite(black) direction as the previous saccade. Bin size is 500ms. Bars show SEM. The blue trace is constructed using 327 fixations from 177 cells examined over 14 fish. The black trace is constructed using 2,864 fixations from 389 cells examined over 16 fish. Source data are provided in a Source Data file.

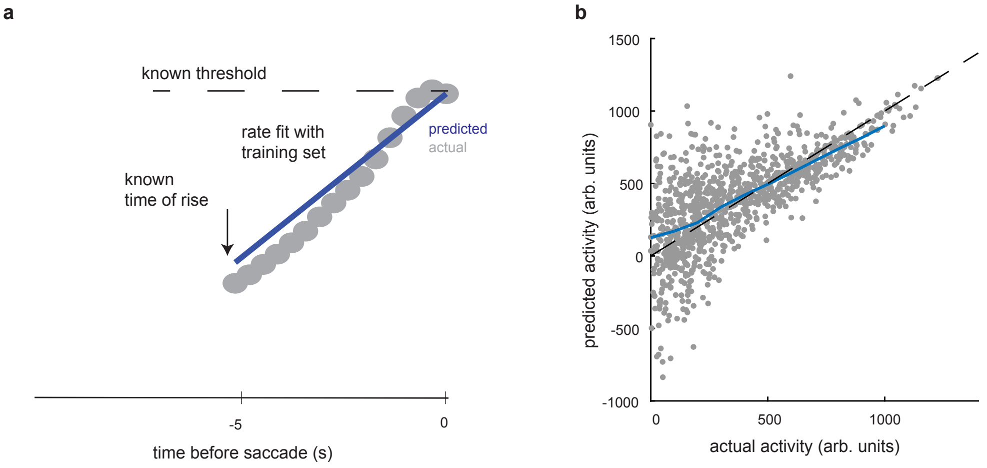

Supplementary Figure 6. Comparison of SR population average pre-saccadic activity with a ramp-to-threshold model. We tested how well SR population average activity fits a model that assumes activity linearly rises from baseline to a threshold value. The model's rate of rise was fit by measuring the average rate of rise of a population average constructed from randomly selected SR cells (the training set, Methods). The model's goodness-of-fit was measured by comparing model activity with a population average constructed from SR cells in the test set. Training set and test set cells are different. (a) Example of population average deconvolved fluorescence from cells in the test set (gray dots) and predicted activity from the ramp-to-threshold model (blue). (b) Quantitative comparison of predicted and actual population average deconvolved fluorescence during SR ramping. Blue line shows median predicted activity (SE = 0.611 (arb. units); n=268,230 predictions derived from 10,000 testing/training sets of population average). Each testing[training] set population average is computed by selecting fixations of equal duration from a set of 2,375 total fixations from 388 cells examined over 16 fish and then randomly selecting 40%[60%] of the selected fixations. Black dashed shows unity line. As seen in (a) the model and actual activity mostly deviate near baseline at values near zero (possibly due to non-linear increases in the population as soon as activity begins to rise). However, the model can reproduce population average activity over a large range of values. Source data are provided in a Source Data file.

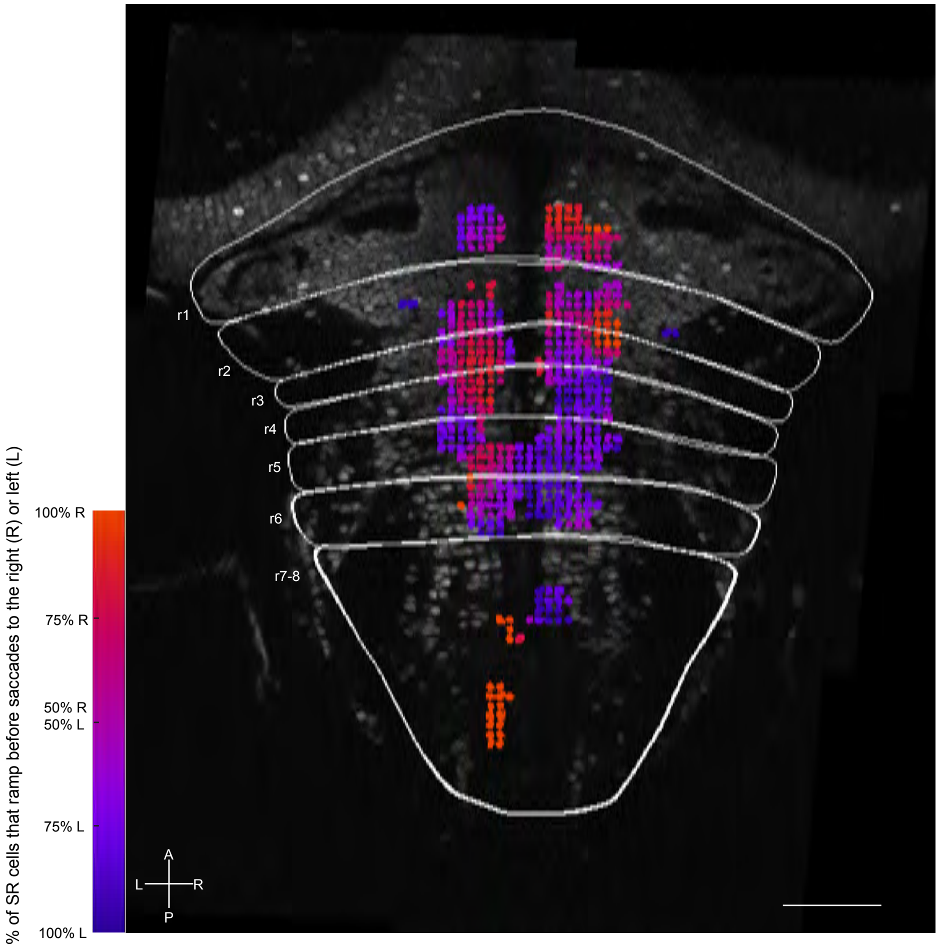

Supplementary Figure 7. Spatial distribution of SR cell direction-preference. Horizontal projection of direction-preference for all SR cells. Black and white background image shows one plane from the bridge brain for visualization purposes. The color of each circle codes the fraction of SR cells that rise before saccades to the right/left and that are registered to a 10 micron radius centered on the circle. No circle is shown if less than five cells are within a 10 micron radius of circle center. Circles with violet colors in-between blue and orange indicate that cells with both left and right preference were registered to that location. The fraction of SR cells that rise before saccades to the left is one minus the fraction of SR cells that rise before saccades to the right. Cells with ipsiversive preference are located in the left/right hemisphere and ramp before saccades to the left/right. Scale bar length is 50 microns. r, rhombomere; A, P, L, R denote anterior, posterior, left and right.

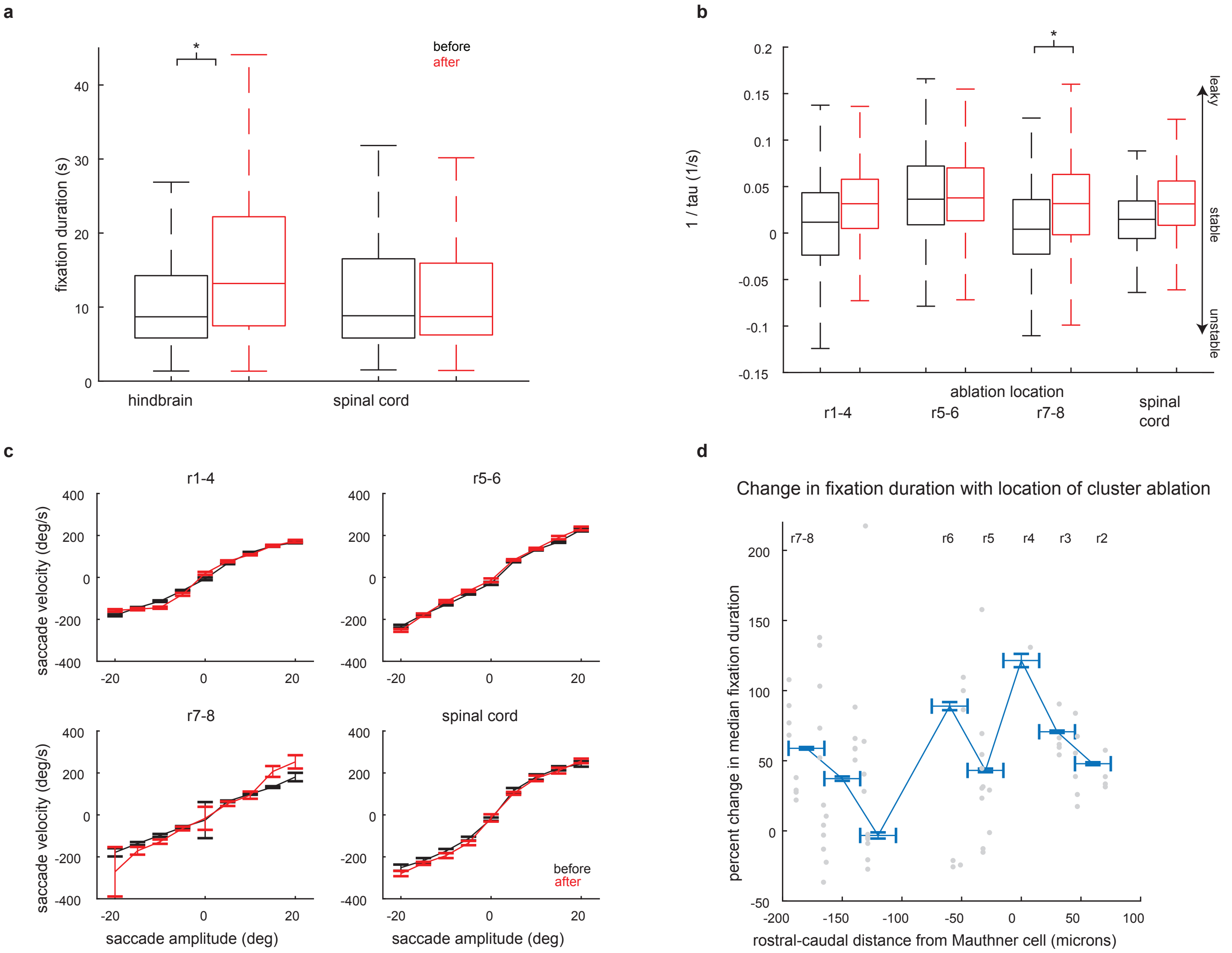

Supplementary Figure 8. Changes in eye-movement metrics following cluster ablations. (a) Fixation durations before (black) and after (red) hindbrain and spinal cord cluster ablations. Left and right eye fixations are combined. Star shows significant differences ($p<0.0001$, two-sample, two-sided t-tests using Bonferroni correction to control for familywise error rate, n = 8,375 fixations before and 3,839 after from 26 fish). (b) Boxplots showing the rate of eye position decay following saccades as a function of ablation location. Eye position decay before (black) and after (red) ablation was measured using an exponential function with time constant tau. Variable number of samples: r1-4 (n=1,308 fixations before, 1,318 fixations after, 6 fish), r5-6 (n=946 fixations before, 1,987 fixations after, 9 fish), r7-8 (n= 3,068 fixations before, 2,209 fixations after, 9 fish), and spinal cord (n=320 fixations before, 1,150 fixations after, 8 fish). For boxplots in A and B, central line shows the median, box limits show the 25th and 75th percentiles, whiskers show 1.5 x interquartile range about upper and lower quartiles. Star shows significant differences ($p<0.0001$, two-sample, two-sided t-tests using Bonferroni correction to control for familywise error rate). (c) Median saccade velocity, grouped by ablation location, before (gray) and after (red) ablations as a function of saccade amplitude (within 5 degree bins). Variable number of samples: r1-4 (n=2,060 saccades before, 1,036 saccades after, 6 fish), r5-6 (n=2,037 saccades before, 1,151 saccades after, 9 fish), r7-8 (n= 4,542 saccades before, 1,819 saccades after, 9 fish), and spinal cord (n=911 saccades before, 1,114 saccades after, 8 fish). Error bars show SEM. (d) Each gray point shows the percent change in median fixation duration following cluster ablation from a single animal (n= 57 randomly selected samples to construct median; n=82 points displayed sampled from 29 fish; see Methods). Blue line shows median +- SEM change across 100 bootstrap replicates (bin size equals 30 microns; n=6,800 samples independently constructed from 29 fish). Bins with less than 10 samples are not plotted. r in (b), (c), (d) stands for rhombomere. Source data are provided in a Source Data file.

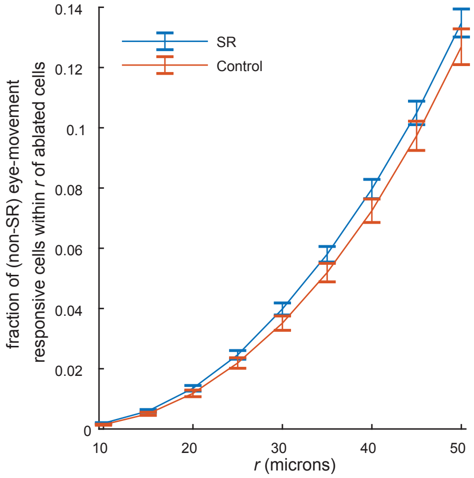

Supplementary Figure 9. Differences in proximity between ablated SR and control cells to (non-SR) eye-movement responsive cells do not explain relative increase in fixation duration after ablating SR cells compared to control. The fraction of (non-SR) eye-movement responsive cells within a sphere of radius, *r*, centered at each ablated SR (blue line; n=48 cells examined over 10 independent experiments) and control (orange line; n=48 cells examined over 10 independent experiments) cell is plotted versus *r*. Only data from single-cell targeted ablations are used. Data are presented as mean values +/- SEM. The center-of-mass location of each ablated SR and control cell was registered to the zBrain atlas reference brain to compare with locations of non-SR, eye-movement responsive cells (Fig 3h). There is no significant difference in the number of nearby eye-movement responsive cells between treated and control groups. Source data are provided in a Source Data file.
