# Supplementary material for: Ramp-to-threshold dynamics in a hindbrain population controls the timing of spontaneous saccades
Source: Nat Commun. 2021 Jul 6;12:4145. doi: 10.1038/s41467-021-24336-w (PMC8260785; doi:10.1038/s41467-021-24336-w)
Supplement: Supplementary file 2 — Reporting Summary [file 41467_2021_24336_MOESM2_ESM.pdf]

Corresponding author(s): Ramirez, Alexandro

Last updated by author(s): Jun 1, 2021

Reporting Summary

Nature Research wishes to improve the reproducibility of the work that we publish. This form provides structure for consistency and transparency in reporting. For further information on Nature Research policies, see our Editorial Policies and the Editorial Policy Checklist.

Statistics

For all statistical analyses, confirm that the following items are present in the figure legend, table legend, main text, or Methods section.

n/a	Confirmed	
☐	☒	The exact sample size (*n*) for each experimental group/condition, given as a discrete number and unit of measurement
☐	☒	A statement on whether measurements were taken from distinct samples or whether the same sample was measured repeatedly
☐	☒	The statistical test(s) used AND whether they are one- or two-sided<br>*Only common tests should be described solely by name; describe more complex techniques in the Methods section.*
☐	☒	A description of all covariates tested
☐	☒	A description of any assumptions or corrections, such as tests of normality and adjustment for multiple comparisons
☐	☒	A full description of the statistical parameters including central tendency (e.g. means) or other basic estimates (e.g. regression coefficient) AND variation (e.g. standard deviation) or associated estimates of uncertainty (e.g. confidence intervals)
☐	☒	For null hypothesis testing, the test statistic (e.g. *F*, *t*, *r*) with confidence intervals, effect sizes, degrees of freedom and *P* value noted<br>*Give P values as exact values whenever suitable.*
☒	☐	For Bayesian analysis, information on the choice of priors and Markov chain Monte Carlo settings
☒	☐	For hierarchical and complex designs, identification of the appropriate level for tests and full reporting of outcomes
☐	☒	Estimates of effect sizes (e.g. Cohen's *d*, Pearson's *r*), indicating how they were calculated

Our web collection on statistics for biologists contains articles on many of the points above.

Software and code

Policy information about availability of computer code

Data collection	Data was collected on a two-photon microscope controlled by ScanImage v3.5 run on Matlab_R2007
Data analysis	We used the CaImAn package run on Matlab_R2018b to extract footprints and fluorescence traces from raw calcium images (https://github.com/flatironinstitute/CaImAn-MATLAB). A description of the parameters we used when running CaImAn is given in the Supplementary Information. We used the BigWarp function from the BigDataViewer FIJI (Version 2.0.0-rc-69/1.52/n) plugin to find points used for registration between ROI coordinates and the H2B-RFP Z-brain reference brain. Calcium traces and simultaneously recorded eye movements were analyzed in MATLAB R2018b using custom-written software which is available here https://github.com/alxdroR/SRNatComm21.git

For manuscripts utilizing custom algorithms or software that are central to the research but not yet described in published literature, software must be made available to editors and reviewers. We strongly encourage code deposition in a community repository (e.g. GitHub). See the Nature Research guidelines for submitting code & software for further information.

Data

Policy information about availability of data

All manuscripts must include a data availability statement. This statement should provide the following information, where applicable:

- Accession codes, unique identifiers, or web links for publicly available datasets
- A list of figures that have associated raw data
- A description of any restrictions on data availability

The calcium and eye movement traces that support the findings of this study are available in figshare with the identifier DOI: https://doi.org/10.6084/m9.figshare.14558064.v1
Data underlying Figs. 1c-e, 2c-e(STAs), 3a-d, 3g, 4e-g, 5a, 5c-d, 5f, 5h-I, 6a-c, 7d-e and Supplementary Figs. 1e, 2a, 2c, 3a, 4a, 4c-d, 4f, 5b-c, 6b, 8, 9 are provided in a Source Data file.

Field-specific reporting

Please select the one below that is the best fit for your research. If you are not sure, read the appropriate sections before making your selection.

- [x] Life sciences
- [ ] Behavioural & social sciences
- [ ] Ecological, evolutionary & environmental sciences

For a reference copy of the document with all sections, see nature.com/documents/nr-reporting-summary-flat.pdf

Life sciences study design

All studies must disclose on these points even when the disclosure is negative.

Sample size	Sample sizes were chosen to be consistent with previously published work in the field (Miri et al., 2011, Nature Neuroscience; Kinkhabwala et al., 2011, PNAS; Ahrens et al. 2012, Nature; Daie et al., 2015, Neuron).
Data exclusions	Animals whose behavior (infrequent or small amplitude saccades) and vitals (blood flow) indicated that animal health was compromised during sample preparation were excluded from all imaging and ablation experiments. These criteria have been used in previous studies.
Replication	The patterns of calcium activity and ablation results reported in the text were observed in more than one animal from at least 2 clutches. The exact number of replicates across animals are reported in the manuscript. All attempts at replication were successful with effect size variability reported in the manuscript.
Randomization	Fish were randomly assigned to experimental and control groups.
Blinding	Blinding is not relevant to results accompanying figures 1-6 since these come from observational studies of neuronal activity and behavior that occurred independent of experimental treatment. The results from the laser ablation experiments were found using software that returns the same result whether or not the experimenter was blinded to control and treatment groups. It was not possible for the experimenter to be blind to treatment and control groups during the experiments since the researcher needed to know this information in order to know where to ablate.

Reporting for specific materials, systems and methods

We require information from authors about some types of materials, experimental systems and methods used in many studies. Here, indicate whether each material, system or method listed is relevant to your study. If you are not sure if a list item applies to your research, read the appropriate section before selecting a response.

Materials & experimental systems

n/a	Involved in the study
☒	☐ Antibodies
☒	☐ Eukaryotic cell lines
☒	☐ Palaeontology and archaeology
☐	☒ Animals and other organisms
☒	☐ Human research participants
☒	☐ Clinical data
☒	☐ Dual use research of concern

Methods

n/a	Involved in the study
☒	☐ ChIP-seq
☒	☐ Flow cytometry
☒	☐ MRI-based neuroimaging

Animals and other organisms

Policy information about studies involving animals; ARRIVE guidelines recommended for reporting animal research

Laboratory animals	Larval Danio Rerio at 7-8 days post-fertilization Tg(HuC:GCaMP6f-H2B; strain cy73-431) kindly provided by Misha Ahren's lab
Wild animals	The study did not involve wild animals
Field-collected samples	The study did not involve samples collected from the field
Ethics oversight	Weill Cornell Medicine's Institutional Animal Care and Use Committee.

Note that full information on the approval of the study protocol must also be provided in the manuscript.
